# Supplementary material for: The genome sequence of the wisent (Bison bonasus)
Source: Gigascience. 2017 Mar 10;6(4):1–5. doi: 10.1093/gigascience/gix016 (PMC5530314; doi:10.1093/gigascience/gix016)
Supplement: GIGA-D-16-00043_Original_Submission.pdf [file gix016_GIGA-D-16-00043_Original_Submission.pdf]

Manuscript

Click here to view linked References

Click here to download Manuscript Wisent Genome Giga Science.docx

Daft genome of European bison (wisent), *Bison bonasus*

Kun Wang[1,6], Lizhong Wang[2,6], Johannes A. Lenstra[3,6], Jianbo Jian[4,6], Quanjun Hu[1,5], Yongzhi Yang[2], Deyong Lai[4], Qiang Qiu[2], Tao Ma[1], Richard Abbott[5], Jianquan Liu[1,2]*

[1]MOE Key Laboratory for Bio-resources and Eco-environment, College of Life Science, Sichuan University, Chengdu, China; [2]State Key Laboratory of Grassland Agro-Ecosystem, College of Life Science, Lanzhou University, Lanzhou, China.[3]Faculty of Veterinary Medicine, Utrecht University, Utrecht, The Netherlands; [4]BGI-Shenzhen, Shenzhen, China; [5]School of Biology, University of St Andrews, St Andrews, Fife KY16 9TH, UK. [6]These authors contributed equally to this work; *Correspondence should be addressed to Ji.L. (liujq@lzu.edu.cn).

Abstract

Background

Wisent, also known as European bison, was rescued approximately 80 years ago from 12 founding individuals. Here, we present a genomic resource built on the basis of a male wisent individual.

Findings

A total of 366 billion base pairs (Gb) of raw reads from whole-genome sequencing of a wisent were generated by the Illumina HiSeq2000 platform. The final genome assembly (2.58 Gb), about 86.5% of the estimated genome size (2.98 Gb), is composed of 29,074 scaffolds with an N50 of 4.7 Mb. 47.3% of the genome is composed of repetitive elements. We identified 22,254 genes and 58,385 non-coding RNA.

Conclusions

We report the first genome sequencing, assembly, and annotation of the wisent. The assembled draft genome will provide a valuable resource for addressing diverse questions of the bovine species.

Keywords

Wisent – Bovine – Genome assembly

Data description

Wisent (*Bison bonasus*), an impressively mighty mammal in Europe, is much larger than its close relatives in the Bovine [1]. In prehistoric Europe, wisent was widely distributed as a major herbivore in broad-leaf forest and/or forest-steppe ecosystems [1]. However, due to unrestricted hunting, plus habitat degradation and fragmentation resulting from increased agricultural activity and forest logging, the last wild animals in the Caucasus went extinct in 1927 [1, 2]. Wisent is now listed as threated by the International Union for Conservation of

Nature [1]. All current wisents in European zoos were rescued approximately 80 years ago from 12 founding individuals.

The wisent sample was collected from tongue of a dead male in the National Park Zuid-Kennerland (The Netherlands). Genomic DNA was isolated using a Qiagen DNA purification kit. Sequencing libraries were constricted with multiple insert sizes (170bp to 20kb) according to the Illumina protocol. For short insert sizes (170 to 800 bp), 6 μg of DNA was fragmented, end-paired and ligated to Illumina paired-end adaptors by following the Illumina protocols. Ligated fragments were size selected at 170, 200, 500 and 800 bp on agarose gels and purified by PCR amplification to yield the corresponding libraries. For long insert sizes (2, 5, 10 and 20 kb) mate-pair library construction, 60 μg of genomic DNA was used; we circularized DNA, digested remaining linear DNA, fragmented circularized DNA, and purified biotinylated DNA and then performed adaptor ligation. All libraries were sequenced on an Illumina HiSeq 2000 platform **(Table S1)**.

For de novo genome assembly, we corrected the reads with short-insert (<2kb) libraries of wisent01 and bison01 using SOAPec [3], a kmer-based error correction software. Using the kmer distribution **(Fig. S1)**, the genome size of wisent was estimated to be 2.98Gb.

The assembly process was conducted in three steps: (i) reads from the same short-insert libraries were assembled with ABySS [4] into distinct contigs on the basis of k-mer overlap information. (ii) reads from the long insert (≥2kp) libraries were aligned to the contig sequence, and the paired end relationships between reads were used to construct scaffolds using SSPACE [5]. (iii) we applied Gapcloser from Short Oligonucleotide Analysis Package (SOAP) [3], which used the paired-end information to retrieve read pairs that had one read well aligned on the contigs and another read located within the gap region, and then performed a local assembly of the collected reads to fill gaps between scaffolds.

De novo assembly yielded a draft wisent assembly with a total length of 2.58 Gb, similar to the 2.66 Gb and 2.65 Gb obtained for the yak and cattle genome, respectively. The N50s of the contigs and scaffolds of wisent were 15 kb and 4.7 Mb, respectively **(Table S2)**. The sequencing depth of 98% of the assembly was more than 20-fold **(Fig. S2)**, ensuring high accuracy at the nucleotide level.

The repetitive regions of wisent sequences were identified with a combination of homology-based and *de novo* approaches. For homology-based repetitive sequences and transposable

elements (TE) listed in Repbase and TE protein database, RepeatMasker [6] and RepeatProteinMask were used. In addition, repeat elements were predicted de novo by Tandem Repeats Finder (TRF) [7], LTR_FINDER [8], PILER [9] and RepeatScout [10] with default parameters. We found 47.3% in the genome of wisent is composed of repetitive elements **(Table S3)**.

We used homology and de novo prediction to identify protein-coding genes. For homology-based gene prediction, protein sequences from six mammals (human, mouse, horse, sheep, cattle, yak) were aligned to the genome of wisent with TBLASTN [11] and every potential gene region, identified and extracted with BLAST2GENE [12] and extended with 5kb in both 5'UTR and 3'UTR. We then applied GeneWise [13] for accurate spliced alignment of extended potential gene region and matching protein sequence. For de novo gene prediction, we applied Augustus [14] and GenScan [15], using parameters trained for wisent and human. We then used EVM [16] to integrate homologues and genes predicted by de novo approaches and generated a consensus gene set. Based on the repeat masked genome, a total of 22,254 genes were predicted to be present in the wisent genome, 98.6% of which had known homologues in protein databases **(Table S4)**. In addition, we also identified 58,385 non-coding RNA in the wisent genome **(Table S5)**.

In summary, we report the first genome sequencing, assembly, and annotation of wisent. The draft genome will provide a valuable resource for studying evolutionary history of bovine species and improving the molecular breeding of bovine.

Acknowledgements

This work was supported by National Natural Science Foundation of China (91331102), the Youth Science and Technology Innovation Team of Sichuan Province (2014TD003), Ministry of Science and Technology of the People's Republic of China (2010DFA34610), International Collaboration 111 Projects of China, Fundamental Research Funds for the Central Universities, 985 and 211 Projects of Sichuan University. We thank Mark Hoyer DVM, Artis Zoo, Amsterdam, for providing the wisent tissue samples.

Availability of supporting data

The assembly and annotation of the wisent genome are available at yak genome database (http://me.lzu.edu.cn/yak). The sequencing reads of each sequencing library have been deposited at NCBI with the Project ID SRS1439150. All supplementary figures and tables are provided in Additional file 1.

References

1. Pucek Z, Belousova IP, Krasinska M, Krasinski ZA, Olech W: **Status survey and conservation action plan. European bison.**; 2004.
2. Bocherens H, Hofman-Kaminska E, Drucker DG, Schmolcke U, Kowalczyk R: **European bison as a refugee species? Evidence from isotopic data on Early Holocene bison and other large herbivores in northern Europe**. *PloS one.* 2015, **10**(2):e0115090.
3. Luo R, Liu B, Xie Y, Li Z, Huang W, Yuan J, He G, Chen Y, Pan Q, Liu Y *et al*: **SOAPdenovo2: an empirically improved memory-efficient short-read de novo assembler**. *Gigascience.* 2012, **1**(1):18.
4. Simpson JT, Wong K, Jackman SD, Schein JE, Jones SJ, Birol I: **ABySS: a parallel assembler for short read sequence data**. *Genome Res.* 2009, **19**(6):1117-1123.
5. Hunt M, Newbold C, Berriman M, Otto TD: **A comprehensive evaluation of assembly scaffolding tools**. *Genome Biol.* 2014, **15**(3):R42.
6. **R. RepeatModeler Open-1.0.** [http://www.repeatmasker.org]
7. Benson G: **Tandem repeats finder: a program to analyze DNA sequences**. *Nucleic Acids Res.* 1999, **27**(2):573-580.
8. Xu Z, Wang H: **LTR_FINDER: an efficient tool for the prediction of full-length LTR retrotransposons**. *Nucleic Acids Res.* 2007, **35**(Web Server issue):W265-268.
9. Edgar RC, Myers EW: **PILER: identification and classification of genomic repeats**. *Bioinformatics.* 2005, **21 Suppl 1**:i152-158.
10. Price AL, Jones NC, Pevzner PA: **De novo identification of repeat families in large genomes**. *Bioinformatics.* 2005, **21 Suppl 1**:i351-358.
11. Camacho C, Coulouris G, Avagyan V, Ma N, Papadopoulos J, Bealer K, Madden TL: **BLAST+: architecture and applications**. *BMC Bioinformatics.* 2009, **10**:421.
12. Suyama M, Torrents D, Bork P: **BLAST2GENE: a comprehensive conversion of BLAST output into independent genes and gene fragments**. *Bioinformatics.* 2004, **20**(12):1968-1970.
13. Birney E, Clamp M, Durbin R: **GeneWise and Genomewise**. *Genome Res.* 2004, **14**(5):988-995.
14. Stanke M, Diekhans M, Baertsch R, Haussler D: **Using native and syntenically mapped cDNA alignments to improve de novo gene finding**. *Bioinformatics.* 2008, **24**(5):637-644.
15. Burge CB, Karlin S: **Finding the genes in genomic DNA**. *Curr Opin Struct Biol.* 1998, **8**(3):346-354.
16. Haas BJ, Salzberg SL, Zhu W, Pertea M, Allen JE, Orvis J, White O, Buell CR, Wortman JR: **Automated eukaryotic gene structure annotation using EVidenceModeler and the Program to Assemble Spliced Alignments**. *Genome Biol.* 2008, **9**(1):R7.

Click here to access/download
Supplementary Material
Wisent Genome Giga Science-S1.docx
