# Supplementary material for: The genome sequence of the wisent (Bison bonasus)
Source: Gigascience. 2017 Mar 10;6(4):1–5. doi: 10.1093/gigascience/gix016 (PMC5530314; doi:10.1093/gigascience/gix016)
Supplement: GIGA-D-16-00043_Revision_1.pdf [file gix016_GIGA-D-16-00043_Revision_1.pdf]

Manuscript

Click here to view linked References

Click here to download Manuscript Wisent Genome Giga Science.docx

The genome sequence of the wisent (*Bison bonasus*)

Kun Wang[1,7], Lizhong Wang[4,7], Johannes A. Lenstra[3,7], Jianbo Jian[4,7], Yongzhi Yang[2], Quanjun Hu[1], Deyong Lai[4], Qiang Qiu[2], Tao Ma[1], Zheng Du[5], Richard Abbott[6], Jianquan Liu[1,2]*

[1]MOE Key Laboratory for Bio-resources and Eco-environment, College of Life Science, Sichuan University, Chengdu, China; [2]State Key Laboratory of Grassland Agro-Ecosystem, College of Life Science, Lanzhou University, Lanzhou, China; [3]Faculty of Veterinary Medicine, Utrecht University, Utrecht, The Netherlands; [4]BGI-Shenzhen, Shenzhen, China; [5]National Supercomputing Center in Shenzhen; [6]School of Biology, University of St Andrews, St Andrews, Fife KY16 9TH, UK. [7]These authors contributed equally to this work; *Correspondence should be addressed to Ji.L. (liujq@lzu.edu.cn).

Abstract

Background

Wisent, also known as European bison, was rescued approximately 80 years ago from 12 founding individuals. Here, we present its draft genome sequence of a male wisent individual.

Findings

A total of 366 billion base pairs (Gb) of raw reads from whole-genome sequencing of a wisent were generated by the Illumina HiSeq2000 platform. The final genome assembly (2.58 Gb), about 86.5% of the estimated genome size (2.98 Gb), is composed of 29,074 scaffolds with an N50 of 4.7 Mb. 47.3% of the genome is composed of repetitive elements. We identified 21,542 genes and 58,385 non-coding RNA. A phylogenetic tree of nuclear genomes reproduces the close relationship if bison and wisent and the sister clades of yak and wisent-bison. We identified 75 genes that has been subject to positive selection in the wisent lineage.

Conclusions

We provide the first genome sequence and gene annotation for the wisent. This will be valuable resources for the conservation of this endangered large mammal and for reconstructing the evolutionary history of the bovine species.

Keywords

Wisent – *Bovini* tribe – Genome assembly

Data description

Background

Wisent (*Bison bonasus*) is an impressively mammal in Europe and one of the largest than its close relatives in the tribe of the *Bovini* [1]. In prehistoric Europe, wisent was widely distributed as a major herbivore in broad-leaf forest and/or forest-steppe ecosystems [1]. However, due to unrestricted hunting, and degradation of habitats by agricultural activity and forest logging, the last wild population in the Caucasus disappeared in 1927 [1, 2]. Wisent is now listed as threatened species by the International Union for Conservation of Nature [1]. All current wisents kept in European zoos and reservations since approximately 80 years ago descend from 12 founding individuals.

Sequencing

The wisent sample was collected from tongue of a dead male in the National Park Zuid-Kennerland (The Netherlands). Genomic DNA was isolated using a Qiagen DNA purification kit. Sequencing libraries with different insert sizes were constructed according to the Illumina protocol. For insert sizes of 170 to 800 bp, 6 μg of DNA was fragmented, end-paired and ligated to Illumina paired-end adaptors. Ligated fragments of 170, 200, 500 and 800 bp were fractionated on agarose gels and purified by PCR amplification to yield the corresponding libraries. For mate-pair library construction with insert sizes of 2, 5, 10 and 20 kb, 60 μg of genomic DNA was and circularized remaining linear DNA was digested. The circularized DNA was fragmented, purified as biotinylated DNA and ligated to adapters. All libraries were sequenced on an Illumina HiSeq 2000 platform **(Table S1)**.

Assembly

For *de novo* genome assembly, we corrected the reads with short-inserts using SOAPec [3], a kmer-based error correction software. Using the kmer distribution **(Fig. S1)**, the genome size of wisent was estimated to be 2.98Gb.

The assembly was performed in three steps: (i) reads from the same short-insert libraries were assembled with ABySS [4] into distinct contigs on the basis of k-mer overlap information; (ii) reads from the long insert (≥2kp) libraries were aligned to the contig sequence and the paired end relationships between reads were used to construct scaffolds using SSPACE [5]; (iii) gaps between scaffolds were closed using Gapcloser from Short Oligonucleotide Analysis Package (SOAP) [3]. This approach used the paired-end information to retrieve read pairs that had one read well aligned to a contig and the other read located within a gap region, and then performed a local assembly of the collected reads in the gap region. This filled 285,642 (47.1%) of gaps and reduced the number of ambiguous bases from 182 to 125 million base pairs.

De novo assembly yielded a draft wisent genome with a length of 2.58 Gb, similar to 2.66 Gb and 2.65 Gb obtained for the yak and taurine cattle genomes, respectively. The N50s of contigs and scaffolds of wisent were 15 kb and 4.7 Mb, respectively **(Table S2)**, similar to the recently published animal genomes (**Table S3**) and the scaffold N50 is much longer than that of yak genome (**Table 1**). To ensure the reliability of our assembly method, we generated another genome assembly with SOAPdenovo and the similar FRCurves [6] proved that our method is acceptable **(Fig. S2)**. The sequencing depth of 98% of the assembly was more than

20-fold **(Fig. S3)**, ensuring a high accuracy at the nucleotide level. To evaluate the integrity of this assembly, we applied BUSCO [7] and CEGMA [8] analyses on the wisent genome and compared with the taurine cattle and yak genomes. BUSCO (**Table S4**) showed that 99% of 3023 single-copy orthologs of the vertebrate lineages were assembled in the wisent genome, which is comparable to percentages for the taurine cattle and yak genomes. CEGMA exhibited similar percent of core eukaryotic genes dataset within these three assemblies (**Table S5**). In addition, mitochondrial DNA sequence with a length of 16,326 bp was recovered by aligning the reads of pair-end libraries to the published wisent mitochondrial sequence and assembling by ABySS.

We mapped the reads from short-insert length libraries to the wisent reference genome with BWA [9] and performed variants calling with GATK [10]. With strict quality control and filtering, we obtained a total of 1.94 million SNVs **(Table S6)** and the heterozygosity rate (0.79×10^{-3}) was similar to that estimated for yak (0.89×10^{-3}) [11]. Moreover, a total of 155,975 insertions and deletions (**Table S7**) were obtained after carefully filtered. Similar to previous studies in yak [12], the InDels in the coding regions exhibit an enrichment for sizes that were multiples of three bases (**Fig. S4**).

Annotation

The repetitive regions of wisent sequences were identified with a combination of homology-based and *de novo* approaches. For homology-based repetitive sequences and transposable elements (TE) listed in Repbase and TE protein database, RepeatMasker [13] and RepeatProteinMask were used. In addition, repeat elements were predicted *de novo* by Tandem Repeats Finder (TRF) [14], LTR_FINDER [15], PILER [16] and RepeatScout [17] with default parameters. We found that 47.3% of our wisent assembly is composed of repetitive elements **(Table S8)**, similar to that in yak [11] **(Fig. S5)**.

We used homology and *de novo* prediction to identify protein-coding genes. For homology-based gene prediction, protein sequences from six mammals (human, mouse, horse, sheep, taurine cattle, yak) were aligned to the genome of wisent with TBLASTN [18]. Every potential gene region were identified and extracted with BLAST2GENE [19] and further extended with 5 kb of 5'UTR and 5 kb of 3'UTR. We then applied GeneWise [20] for accurately aligning the extended potential gene region and matching the protein sequences. We used Augustus [21] and GenScan [22] for *de novo* gene prediction based on the

parameters trained for wisent and human. We then used EVM [23] to integrate homologues and genes predicted by the *de novo* approach and generated a consensus gene set. A total of 21,542 genes were predicted to be present in the repeat-masked wisent genome, 73% and 68% of which were annotated by Gene Ontology [24] and KEGG [25] respectively. In addition, we identified 58,385 non-coding RNA in the wisent genome **(Table S9)**.

Compared the gene set of wisent to yak and taurine cattle, there were no significant differences between the gene number and the distribution of gene length, exon length etc. **(Table 1, Fig. S6)**. We further compared the gene composition of wisent to yak and taurine cattle with the latest KEGG database and we found no pathway show significant difference except two pathways, ko04740 (Olfactory transduction) and ko03010 (Ribosome), contain significant more genes in yak. These results indicated that our gene prediction was reliable and the gene composition were conservative in these three species.

Comparative analysis

We downloaded the nuclear genomes of including yak, taurine cattle, zebu cattle, bison and water buffalo from GenBank (GCA_000298355.1, GCA_000003205.4, GCA_000247795.2, GCA_000754665.1, GCA_000471725.1) [11, 26-28] and constructed a synteny alignment spanning1,055 Mb across all genomes (**Fig. S7**). We found an average nuclear distance (percent of different base pairs in the synteny regions) between wisent and bison of 0.47% (**Fig. S8**), less than half of the distance between wisent and taurine cattle (0.98%) or between taurine cattle and bison (0.93%). Phylogenetic relationship reconstructed with ExaML [29] (**Fig. S9**) also suggested the sister relationship between wisent and bison. The speciation pattern and evolutionary diversification of the bovine species need further detailed examination based on more population genomic data through re-sequencing genomes of more individuals.

To predict the species-specific and commonly shared genes in wisent and relative species, we used orthoMCL [30] to define the clusters of orthologous genes. We downloaded gene set of 6 additional species (human, dog, horse, sheep, taurine cattle, yak) from Ensembl [31] and related database [32]. In total, we identified 12,358 homologous gene families shared by 7 species **(Table S10)**: 272 gene families were specific to wisent and yak and 58 was only in wisent **(Fig. S10)**. We finally used the branch-site likelihood ratio test [33] to identify positively selected genes (PSG) in the wisent lineage compared with others in the Bovine.

We identified 75 PSGs, which are enriched in tissue remodeling and ion transport (**Table S11, S12**).

The genomic resources describe in this report will be useful for investigating evolutionary histories of the bovine species and also be relevant for the conservation of the wisent.

Acknowledgements

This work was supported by the Youth Science and Technology Innovation Team of Sichuan Province (2014TD003), National Natural Science Foundation of China (91331102), Ministry of Science and Technology of the People's Republic of China (2010DFA34610), Shenzhen Industrial Designation Services Cloud Platform (GGJS20150429172906635), International Collaboration 111 Projects of China, Fundamental Research Funds for the Central Universities, 985 and 211 Projects of Sichuan University. We thank Mark Hoyer DVM, Artis Zoo, Amsterdam, for providing the wisent tissue samples.

Availability of supporting data

The assembly and annotation of the wisent genome are available at yak genome database (http://me.lzu.edu.cn/yak). The sequencing reads of each sequencing library have been deposited at NCBI with the Project ID: PRJNA321590, Sample ID: SRS1439150. Supplementary figures and tables are provided in Additional file 1.

References

1. Pucek Z, Belousova IP, Krasinska M, Krasinski ZA, Olech W: **Status survey and conservation action plan. European bison.**; 2004.
2. Bocherens H, Hofman-Kaminska E, Drucker DG, Schmolcke U, Kowalczyk R: **European bison as a refugee species? Evidence from isotopic data on Early Holocene bison and other large herbivores in northern Europe**. *PloS one* 2015, **10**(2):e0115090.
3. Luo R, Liu B, Xie Y, Li Z, Huang W, Yuan J, He G, Chen Y, Pan Q, Liu Y *et al*: **SOAPdenovo2: an empirically improved memory-efficient short-read de novo assembler**. *Gigascience* 2012, **1**(1):18.
4. Simpson JT, Wong K, Jackman SD, Schein JE, Jones SJ, Birol I: **ABySS: a parallel assembler for short read sequence data**. *Genome research* 2009, **19**(6):1117-1123.
5. Hunt M, Newbold C, Berriman M, Otto TD: **A comprehensive evaluation of assembly scaffolding tools**. *Genome biology* 2014, **15**(3):R42.

6. Vezzi F, Narzisi G, Mishra B: **Reevaluating assembly evaluations with feature response curves: GAGE and assemblathons**. *PloS one* 2012, **7**(12):e52210.
7. Simao FA, Waterhouse RM, Ioannidis P, Kriventseva EV, Zdobnov EM: **BUSCO: assessing genome assembly and annotation completeness with single-copy orthologs**. *Bioinformatics* 2015, **31**(19):3210-3212.
8. Parra G, Bradnam K, Ning Z, Keane T, Korf I: **Assessing the gene space in draft genomes**. *Nucleic acids research* 2009, **37**(1):289-297.
9. Li H: **Aligning sequence reads, clone sequences and assembly contigs with BWA-MEM**. *arXiv:13033997v2* 2013 **q-bio.GN**.
10. DePristo MA, Banks E, Poplin R, Garimella KV, Maguire JR, Hartl C, Philippakis AA, del Angel G, Rivas MA, Hanna M *et al*: **A framework for variation discovery and genotyping using next-generation DNA sequencing data**. *Nat Genet* 2011, **43**(5):491-498.
11. Qiu Q, Zhang G, Ma T, Qian W, Wang J, Ye Z, Cao C, Hu Q, Kim J, Larkin DM *et al*: **The yak genome and adaptation to life at high altitude**. *Nat Genet* 2012, **44**(8):946-949.
12. Wang K, Hu Q, Ma H, Wang L, Yang Y, Luo W, Qiu Q: **Genome-wide variation within and between wild and domestic yak**. *Molecular ecology resources* 2014, **14**(4):794-801.
13. **R. RepeatModeler Open-1.0.** [http://www.repeatmasker.org]
14. Benson G: **Tandem repeats finder: a program to analyze DNA sequences**. *Nucleic acids research* 1999, **27**(2):573-580.
15. Xu Z, Wang H: **LTR_FINDER: an efficient tool for the prediction of full-length LTR retrotransposons**. *Nucleic acids research* 2007, **35**(Web Server issue):W265-268.
16. Edgar RC, Myers EW: **PILER: identification and classification of genomic repeats**. *Bioinformatics* 2005, **21 Suppl 1**:i152-158.
17. Price AL, Jones NC, Pevzner PA: **De novo identification of repeat families in large genomes**. *Bioinformatics* 2005, **21 Suppl 1**:i351-358.
18. Camacho C, Coulouris G, Avagyan V, Ma N, Papadopoulos J, Bealer K, Madden TL: **BLAST+: architecture and applications**. *BMC Bioinformatics* 2009, **10**:421.
19. Suyama M, Torrents D, Bork P: **BLAST2GENE: a comprehensive conversion of BLAST output into independent genes and gene fragments**. *Bioinformatics* 2004, **20**(12):1968-1970.
20. Birney E, Clamp M, Durbin R: **GeneWise and Genomewise**. *Genome research* 2004, **14**(5):988-995.
21. Stanke M, Diekhans M, Baertsch R, Haussler D: **Using native and syntenically mapped cDNA alignments to improve de novo gene finding**. *Bioinformatics* 2008, **24**(5):637-644.
22. Burge CB, Karlin S: **Finding the genes in genomic DNA**. *Curr Opin Struct Biol* 1998, **8**(3):346-354.
23. Haas BJ, Salzberg SL, Zhu W, Pertea M, Allen JE, Orvis J, White O, Buell CR, Wortman JR: **Automated eukaryotic gene structure annotation using EVidenceModeler and the Program to Assemble Spliced Alignments**. *Genome biology* 2008, **9**(1):R7.
24. **Gene Ontology Consortium: going forward**. *Nucleic acids research* 2015, **43**(Database issue):D1049-1056.
25. Kanehisa M, Sato Y, Kawashima M, Furumichi M, Tanabe M: **KEGG as a reference resource for gene and protein annotation**. *Nucleic acids research* 2016, **44**(D1):D457-462.
26. Zimin AV, Delcher AL, Florea L, Kelley DR, Schatz MC, Puiu D, Hanrahan F, Pertea G, Van Tassell CP, Sonstegard TS *et al*: **A whole-genome assembly of the domestic cow, Bos taurus**. *Genome biology* 2009, **10**(4):R42.
27. Canavez FC, Luche DD, Stothard P, Leite KR, Sousa-Canavez JM, Plastow G, Meidanis J, Souza MA, Feijao P, Moore SS *et al*: **Genome sequence and assembly of Bos indicus**. *The Journal of heredity* 2012, **103**(3):342-348.
28. Dobson LK: **Sequencing the Genome of the North American Bison**. *Doctoral dissertation* 2015(Available electronically from http://hdl.handle.net/1969.1/155759).
29. Kozlov AM, Aberer AJ, Stamatakis A: **ExaML version 3: a tool for phylogenomic analyses on supercomputers**. *Bioinformatics* 2015, **31**(15):2577-2579.
30. Li L, Stoeckert CJ, Jr., Roos DS: **OrthoMCL: identification of ortholog groups for eukaryotic genomes**. *Genome research* 2003, **13**(9):2178-2189.

31. Yates A, Akanni W, Amode MR, Barrell D, Billis K, Carvalho-Silva D, Cummins C, Clapham P, Fitzgerald S, Gil L *et al*: **Ensembl 2016**. *Nucleic acids research* 2016, **44**(D1):D710-716.

32. Hu Q, Ma T, Wang K, Xu T, Liu J, Qiu Q: **The Yak genome database: an integrative database for studying yak biology and high-altitude adaption**. *Bmc Genomics* 2012, **13**:600.

33. Yang Z: **PAML 4: phylogenetic analysis by maximum likelihood**. *Molecular biology and evolution* 2007, **24**(8):1586-1591.

Table 1. Comparison of genome assembly and gene annotation in wisent, yak and taurine cattle

Species	Assembled genome size (Mb)	Scaffold N50 (Mb)	Contig N50 (kb)	Gene number	Mean gene length (bp)
Wisent	2,575.96	4.69	14.53	21,542	31,458.00
Yak	2,656.77	1.40	20.45	22,282	29,106.90
Taurine Cattle	2,673.16	105.98	88.13	20,526	40,944.10

Additional files

Figure S1: Kmer distribution in wisent.

Figure S2: FRCurve of two genome assemblies.

Figure S3: Sequencing depth of the assembled wisent genome.

Figure S4: Counts of InDels in coding regions.

Figure S5: Comparison of the composition of repetitive elements in wisent and yak.

Figure S6: Comparison of gene length, intron length, exon length and exon number between taurine cattle, wisent and yak.

Figure S7: Synteny relationship of wisent and taurine cattle in chromosome 1.

Figure S8: Divergence of American-European bison and taurine–zebu cattle.

Figure S9: Phylogeny relationship of wisent in *Bovini* tribe.

Figure S10: Venn diagram of gene families within five species.

Table S1: Summary of sequenced reads.

Table S2: Summary statistics of the genome assembly of wisent.

Table S3: Assembly statistics from published animal genomes generated after 2012.

Table S4: Summary of BUSCO analysis by counting matches to 3023 single-copy orthologs.

Table S5: Summary of CEGMA analysis.

Table S6. The distribution of SNVs in the wisent genome.

Table S7. The distribution of InDels in the wisent genome.

Table S8: Summary statistics of interspersed repeat regions in wisent.

Table S9: Summary statistics of non-coding RNAs in wisent.

Table S10: Summary statistics of gene families in 7 species.

Table S11: Genes subject to positive selection in wisent.

Table S12: Enriched gene ontology of positively selected genes.

Additional file 1

Click here to access/download
Supplementary Material
Wisent Genome Giga Science-Additional file 1.docx

Dear Editor，

Thank you very much for returning my manuscript GIGA-D-16-00043 entitled “The genome sequence of the wisent (*Bison bonasus*)” along with the excellent comments from the communicating editor and the referees.

We are grateful for the constructive and thoughtful comments from the reviewers. Their suggestions have undoubtedly helped to improve the manuscript greatly. We have revised the manuscript according to their suggestions and comments, and responded point by point to their comments.

We submit here the revised manuscript and hope that the revised manuscript is more suitable for the publication in Giga Science. If you have any questions, please do not hesitate to contact the corresponding author at any time.

Thank you again for your time and efforts in handling our manuscript.

Best wishes,

Jianquan Liu
MOE Key Laboratory for Bio-resources and Eco-environment,
College of Life Science,
Sichuan University,
Chengdu, China.
Email: liujq@lzu.edu.cn
