# Supplementary material for: The genome sequence of the wisent (Bison bonasus)
Source: Gigascience. 2017 Mar 10;6(4):1–5. doi: 10.1093/gigascience/gix016 (PMC5530314; doi:10.1093/gigascience/gix016)
Supplement: GIGA-D-16-00043_Revision_3.pdf [file gix016_GIGA-D-16-00043_Revision_3.pdf]

Manuscript

Click here to download Manuscript Wisent Genome-Giga Science-Manuscript.docx

The genome sequence of the wisent (*Bison bonasus*)

Kun Wang[1,7], Lizhong Wang[4,7], Johannes A. Lenstra[3,7], Jianbo Jian[4,7], Yongzhi Yang[2], Quanjun Hu[1], Deyong Lai[4], Qiang Qiu[2], Tao Ma[1], Zheng Du[5], Richard Abbott[6], Jianquan Liu[1,2]*

[1]MOE Key Laboratory for Bio-resources and Eco-environment, College of Life Science, Sichuan University, Chengdu, China; [2]State Key Laboratory of Grassland Agro-Ecosystem, College of Life Science, Lanzhou University, Lanzhou, China; [3]Faculty of Veterinary Medicine, Utrecht University, Utrecht, The Netherlands; [4]BGI-Shenzhen, Shenzhen, China; [5]National Supercomputing Center in Shenzhen; [6]School of Biology, University of St Andrews, St Andrews, Fife KY16 9TH, UK. [7]These authors contributed equally to this work; *Correspondence should be addressed to Ji.L. (liujq@lzu.edu.cn).

Abstract

Background

The wisent, also known as the European bison, was rescued from extinction approximately 80 years ago through the conservation of 12 individuals. Here, we present the draft genome sequence of a male wisent individual descended from this founding stock.

Findings

A total of 366 billion base pairs (Gb) of raw reads from whole-genome sequencing of this wisent were generated using the Illumina HiSeq2000 platform. The final genome assembly (2.58 Gb) is composed of 29,074 scaffolds with an N50 of 4.7 Mb. 47.3% of the genome is composed of repetitive elements. We identified 21,542 genes and 58,385 non-coding RNAs. A phylogenetic tree based on nuclear genomes indicated sister relationships between bison and wisent and between the wisent-bison clade and yak. For 75 genes we obtained evidence of positive evolution in the wisent lineage.

Conclusions

We provide the first genome sequence and gene annotation for the wisent. The availability of these resources will be of value for the future conservation of this endangered large mammal and for reconstructing the evolutionary history of the *Bovini* tribe.

Keywords

Wisent – *Bovini* tribe – Genome assembly

Data description

Background

Wisent (*Bison bonasus*), also known as the European bison, is an impressive mammal of the tribe *Bovini* [1]. In prehistoric Europe, wisent was widely distributed as a major herbivore in broad-leaf forest and/or forest-steppe ecosystems [1]. However, due to unrestricted hunting, and degradation of habitats by agricultural activity and forest logging, the last wild population in the Caucasus disappeared in 1927 [1, 2]. Wisent is now listed as a threatened species by the International Union for Conservation of Nature [1]. All current wisents kept in European zoos and reservations since approximately 80 years ago descend from 12 founding individuals.

Sequencing

The wisent sample analysed was collected from the tongue of a male calf that died in the National Park Zuid-Kennemerland (The Netherlands) in 2014. Genomic DNA was isolated using a Qiagen DNA purification kit. Sequencing libraries with different insert sizes were constructed according to the Illumina protocol. For insert sizes of 170 to 800 bp, 6 μg of DNA was fragmented, end-paired and ligated to Illumina paired-end adaptors. Ligated fragments of 170, 200, 500 and 800 bp were fractionated on agarose gels and purified by PCR amplification to yield the corresponding libraries. For mate-pair library construction with insert sizes of 2, 5, 10 and 20 kb, 60 μg of genomic DNA was used and circularized, while remaining linear DNA was digested. The circularized DNA was fragmented, purified as biotinylated DNA and ligated to adapters. All libraries were sequenced on an Illumina HiSeq 2000 platform **(Table S1)**.

Assembly

For *de novo* genome assembly, we corrected the reads with short-inserts using SOAPec [3], a kmer-based error correction software. On the basis of the k-mer distribution **(Fig. S1)**, the genome size of wisent was estimated to be 2.98Gb.

The assembly was performed in three steps: (i) reads from the same short-insert libraries were assembled with ABySS [4] into distinct contigs on the basis of k-mer overlap information; (ii) reads from the long insert (≥2kb) libraries were aligned to the contig sequence (longer than 500bp) and the paired end relationships between reads were used to construct scaffolds using SSPACE [5]; (iii) gaps between scaffolds were closed using

Gapcloser from the Short Oligonucleotide Analysis Package (SOAP) [3]. This approach used the paired-end information to retrieve read pairs that had one read well aligned to a contig and the other read located within a gap region, and then performed a local assembly of the collected reads in the gap region. This filled 285,642 (47.1%) of gaps and reduced the number of ambiguous bases from 182 to 125 million base pairs.

De novo assembly yielded a draft wisent genome with a length of 2.58 Gb, slightly less than 2.66 Gb and 2.65 Gb obtained for the yak and taurine cattle genomes, respectively, which might be the result of removing contigs less than 500bp in the second step of assembly. The N50s of contigs and scaffolds of wisent were 15 kb and 4.7 Mb, respectively **(Table S2)**, similar to those recorded for other recently published animal genomes (**Table S3**), however the scaffold N50 was much longer than that of the yak genome (**Table 1**). In addition, the mitochondrial DNA sequence with a length of 16,326 bp was recovered by aligning reads of pair-end libraries to the published wisent mitochondrial sequence and assembling by ABySS. This differed only slightly (0.13%) from that obtained from previous mtDNA data [6] (**Fig. S2**).

We examined the quality of the genome assembly by considering base-level accuracy, integrity and continuity, respectively. The sequencing depth of 98% of the assembly was more than 20-fold **(Fig. S3)**, ensuring high accuracy at the nucleotide level. To assess the integrity of our assembly, we carried out BUSCO [7] and CEGMA [8] analyses on the wisent genome. The BUSCO score, using BUSCO gene prediction, was 83.4% (**Table S4**; 94% complete matches if using our annotation) and the CEGMA score was 68.15% (**Table S5**). These scores are comparable to those for taurine cattle (both UMD3.1 and Btau4.6.1) and yak genomes. Finally, to evaluate the trade-off between the contiguity and correctness of our assembly, we applied the FRC method [9], which is based on a prediction of assembly correctness by identifying on each de novo assembled scaffold, 'features' representing potential errors or complications during the assembly process. The FRCurve was calculated on six assemblies (**Fig. S4)**: the wisent genome assembly generated with SSPACE, an alternative wisent genome assembly generated with SOAPdenovo, the UMD3.1 and Btau4.6.1 cattle genome assemblies, the OAR31 sheep assembly and the V1.1 yak assembly, respectively. The similar FRCurves of the two wisent genome assemblies indicate that the SSPACE version of the wisent genome did not lose more correctness than SOAPdenovo, although the scaffold N50 size of SSPACE version is 10 times larger (4.69 instead of 0.47Mb). However, we note that UMD3.1 genome assembly, generated using a combination

of BAC-by-BAC hierarchical and whole-genome shotgun sequencing methods, generated a better FRCurve than the other five assemblies.

We mapped the reads from short-insert length libraries to the wisent reference genome with BWA [10] and performed variant calling with GATK [11]. With strict quality control and filtering, we obtained a total of 1.94 million SNVs **(Table S6)** and noted that heterozygosity rate (0.79×10^{-3}) was similar to that estimated for yak (0.89×10^{-3}) [12]. Moreover, a total of 155,975 insertions and deletions (**Table S7**) were obtained. Similar to previous studies in yak [13], the InDels in the coding regions exhibit an enrichment for sizes that were multiples of three bases (**Fig. S5**).

Annotation

The repetitive regions of wisent sequences were identified with a combination of homology-based and *de novo* approaches. For homology-based repetitive sequences and transposable elements (TE) listed in Repbase and TE protein database, RepeatMasker [14] and RepeatProteinMask were used. In addition, repeat elements were *de novo* predicted by Tandem Repeats Finder (TRF) [15], LTR_FINDER [16], PILER [17] and RepeatScout [18] with default parameters. We found that 47.3% of our wisent assembly is composed of repetitive elements **(Table S8)**, similar to that in yak [12] **(Fig. S6)**.

We used homology and *de novo* prediction to identify protein-coding genes. For homology-based gene prediction, protein sequences from six mammals (human, mouse, horse, sheep, taurine cattle, yak) were aligned to the wisent genome with TBLASTN [19]. Potential gene regions were identified and extracted with BLAST2GENE [20] and further extended with 5 kb of 5'UTR and 5 kb of 3'UTR. We then applied GeneWise [21] for accurately aligning the extended potential gene region and matching the protein sequences. We used Augustus [22] and GenScan [23] for *de novo* gene prediction based on the parameters trained for wisent and human. We then used EVM [24] to integrate homologues and genes predicted by the *de novo* approach and generated a consensus gene set. A total of 21,542 genes were predicted to be present in the repeat-masked wisent genome, 73% and 68% of which were annotated by Gene Ontology [25] and KEGG [26] respectively. In addition, we identified 58,385 non-coding RNAs in the wisent genome **(Table S9)**.

Comparing the wisent genome with those for yak and taurine cattle, we found no significant differences in number of genes, gene length distribution and exon length distribution **(Table**

1, Fig. S7). We further compared the gene compositions of wisent, yak and taurine cattle to the latest KEGG database. No pathway showed a significant difference between the three species except for pathways, ko04740 (Olfactory transduction) and ko03010 (Ribosome), which contained significantly more genes in yak. These results indicate that our gene prediction was reliable and that gene composition was conserved in the three species.

Comparative analysis

In order to identify any large-scale variations between the assembled sequences of wisent and cattle (autosomes and X chromosome from UMD3.1, Y chromosome from Btau4.6.1), we performed synteny analysis with 'last' [27]. A total of 2.22 Gb 1:1 alignment, covered 83% of cattle genome and 86% of wisent genome, was generated (**Fig. S8, table S10**). The sequences of cattle autosomes were well covered (average value = 85%) with the synteny alignment while only 69% and 2% were covered for chromosomes X and Y, respectively. The scaffolds of the wisent genome aligned to the sex chromosomes were also more fragmented. Although most scaffolds in wisent could be well aligned to cattle chromosomes, a significant number of inter-chromosomal rearrangements were present between two species (**Fig. S8**). We then summarized the synteny result of scaffolds larger than 500kb and found that on average 83% of a scaffold could be aligned to one cattle chromosome, while at most 0.3% of the scaffold were aligned to more than two cattle chromosomes (**Fig. S9a**). By comparing wisent and cattle genomes we identified 13,495 inter-chromosomal, intra-chromosomal or inversion breakpoints (edge of transposition events), (**Table S10**), which may be caused by errors in assembly of both genomes, errors of synteny alignments (false positive and false negative) or real transposition events between wisent and cattle. However, it is difficult to distinguish between such possible artifactual and real effects. The breakpoint distributions were significantly enriched in repeat regions, (**Fig S10a**), which are sensitive to rearrangements but also to assembly or alignment errors. Longer scaffolds were found to harbor fewer breakpoints (**Fig. S10b**), reflecting the complexity and often problematic assembly of short scaffolds. Single molecule sequencing with unbiased long reads will be ideal for identifying large-scale variations.

The synteny alignments within six species (yak: GCA_000298355.1, zebu cattle: GCA_000247795.2, bison: GCA_000754665.1, water buffalo: GCA_000471725.1) [12, 28-30] were generated with a combination of 'last' and 'multiZ' [31] (**Table S11**). An average nuclear distance (percent of different base pairs in the synteny regions) between wisent and

bison was 0.37% (**Fig. S11, Table S12**), which is less than that between wisent and taurine cattle (0.93%) or between taurine cattle and bison (0.93%). Phylogenetic relationships reconstructed with Examl [32] (**Fig. S12**) confirmed the sister relationship between wisent and bison, and also a sister relationship between the wisent-bison clade and yak. A more detailed resolution of speciation events and evolutionary histories within the *Bovini* tribe awaits further analyses following the sequencing of genomes of additional individuals of member species.

To predict the species-specific and commonly shared genes in wisent and other species, we used orthoMCL [33] to define clusters of orthologous genes. For this, we downloaded the gene sets of 6 additional species (human, dog, horse, sheep, taurine cattle, yak) from Ensembl [34] and related database [35]. In total, we identified 12,358 homologous gene families shared by wisent and the 6 species **(Table S13)**, 272 gene families that were specific to wisent and yak, and 58 specific only to wisent **(Fig. S13)**. We finally used the branch-site likelihood ratio test [36] to identify positively selected genes (PSG) in the wisent lineage compared with others in the *Bovini* tribe. We identified 75 PSGs, which are enriched in tissue remodeling and ion transport (**Table S14, S15**).

We conclude that the genomic resources described in this report will be useful for investigating the evolutionary histories of the *Bovini* tribe and will have relevance for the future conservation of wisent.

Acknowledgements

This work was supported by the Youth Science and Technology Innovation Team of Sichuan Province (2014TD003), Shenzhen Industrial Designation Services Cloud Platform (GGJS20150429172906635), International Collaboration 111 Projects of China, Fundamental Research Funds for the Central Universities, 985 and 211 Projects of Sichuan University. We thank Mark Hoyer DVM, Artis Zoo, Amsterdam, for providing the wisent tissue samples.

Availability and requirements

- Project name: Assembly pipeline used in the wisent genome paper.
- Project home page: https://github.com/wk8910/assemble_pipeline/
- Operating systems: Unix

- Programming language: Python
- License: Mozilla Public License Version 2.0 (MPLv2)

Availability of supporting data

The sequencing reads of each sequencing library have been deposited at NCBI with the Project ID: PRJNA321590, Sample ID: SRS1439150. The assembly and annotation of the wisent genome are also available in the *GigaScience* GigaDB database[37] and at the yak genome database (http://me.lzu.edu.cn/yak). The assembly pipeline and commands used in this work are available at GitHub (https://github.com/wk8910/assemble_pipeline/). Supplementary figures and tables are provided in Additional file 1.

References

1. Pucek Z, Belousova IP, Krasinska M, Krasinski ZA, Olech W: **Status survey and conservation action plan. European bison.**; 2004.
2. Bocherens H, Hofman-Kaminska E, Drucker DG, Schmolcke U, Kowalczyk R: **European bison as a refugee species? Evidence from isotopic data on Early Holocene bison and other large herbivores in northern Europe**. *PloS one* 2015, **10**(2):e0115090.
3. Luo R, Liu B, Xie Y, Li Z, Huang W, Yuan J, He G, Chen Y, Pan Q, Liu Y *et al*: **SOAPdenovo2: an empirically improved memory-efficient short-read de novo assembler**. *Gigascience* 2012, **1**(1):18.
4. Simpson JT, Wong K, Jackman SD, Schein JE, Jones SJ, Birol I: **ABySS: a parallel assembler for short read sequence data**. *Genome research* 2009, **19**(6):1117-1123.
5. Hunt M, Newbold C, Berriman M, Otto TD: **A comprehensive evaluation of assembly scaffolding tools**. *Genome biology* 2014, **15**(3):R42.
6. Stamatakis A: **RAxML-VI-HPC: maximum likelihood-based phylogenetic analyses with thousands of taxa and mixed models**. *Bioinformatics* 2006, **22**(21):2688-2690.
7. Simao FA, Waterhouse RM, Ioannidis P, Kriventseva EV, Zdobnov EM: **BUSCO: assessing genome assembly and annotation completeness with single-copy orthologs**. *Bioinformatics* 2015, **31**(19):3210-3212.
8. Parra G, Bradnam K, Ning Z, Keane T, Korf I: **Assessing the gene space in draft genomes**. *Nucleic acids research* 2009, **37**(1):289-297.
9. Vezzi F, Narzisi G, Mishra B: **Reevaluating assembly evaluations with feature response curves: GAGE and assemblathons**. *PloS one* 2012, **7**(12):e52210.
10. Li H: **Aligning sequence reads, clone sequences and assembly contigs with BWA-MEM**. *arXiv:13033997v2* 2013 **q-bio.GN**.
11. DePristo MA, Banks E, Poplin R, Garimella KV, Maguire JR, Hartl C, Philippakis AA, del Angel G, Rivas MA, Hanna M *et al*: **A framework for variation discovery and genotyping using next-generation DNA sequencing data**. *Nat Genet* 2011, **43**(5):491-498.
12. Qiu Q, Zhang G, Ma T, Qian W, Wang J, Ye Z, Cao C, Hu Q, Kim J, Larkin DM *et al*: **The yak genome and adaptation to life at high altitude**. *Nat Genet* 2012, **44**(8):946-949.
13. Wang K, Hu Q, Ma H, Wang L, Yang Y, Luo W, Qiu Q: **Genome-wide variation within and between wild and domestic yak**. *Molecular ecology resources* 2014, **14**(4):794-801.
14. **R. RepeatModeler Open-1.0.** [http://www.repeatmasker.org/]

15. Benson G: **Tandem repeats finder: a program to analyze DNA sequences**. *Nucleic acids research* 1999, **27**(2):573-580.
16. Xu Z, Wang H: **LTR_FINDER: an efficient tool for the prediction of full-length LTR retrotransposons**. *Nucleic acids research* 2007, **35**(Web Server issue):W265-268.
17. Edgar RC, Myers EW: **PILER: identification and classification of genomic repeats**. *Bioinformatics* 2005, **21 Suppl 1**:i152-158.
18. Price AL, Jones NC, Pevzner PA: **De novo identification of repeat families in large genomes**. *Bioinformatics* 2005, **21 Suppl 1**:i351-358.
19. Camacho C, Coulouris G, Avagyan V, Ma N, Papadopoulos J, Bealer K, Madden TL: **BLAST+: architecture and applications**. *BMC Bioinformatics* 2009, **10**:421.
20. Suyama M, Torrents D, Bork P: **BLAST2GENE: a comprehensive conversion of BLAST output into independent genes and gene fragments**. *Bioinformatics* 2004, **20**(12):1968-1970.
21. Birney E, Clamp M, Durbin R: **GeneWise and Genomewise**. *Genome research* 2004, **14**(5):988-995.
22. Stanke M, Diekhans M, Baertsch R, Haussler D: **Using native and syntenically mapped cDNA alignments to improve de novo gene finding**. *Bioinformatics* 2008, **24**(5):637-644.
23. Burge CB, Karlin S: **Finding the genes in genomic DNA**. *Curr Opin Struct Biol* 1998, **8**(3):346-354.
24. Haas BJ, Salzberg SL, Zhu W, Pertea M, Allen JE, Orvis J, White O, Buell CR, Wortman JR: **Automated eukaryotic gene structure annotation using EVidenceModeler and the Program to Assemble Spliced Alignments**. *Genome biology* 2008, **9**(1):R7.
25. **Gene Ontology Consortium: going forward**. *Nucleic acids research* 2015, **43**(Database issue):D1049-1056.
26. Kanehisa M, Sato Y, Kawashima M, Furumichi M, Tanabe M: **KEGG as a reference resource for gene and protein annotation**. *Nucleic acids research* 2016, **44**(D1):D457-462.
27. Kielbasa SM, Wan R, Sato K, Horton P, Frith MC: **Adaptive seeds tame genomic sequence comparison**. *Genome research* 2011, **21**(3):487-493.
28. Zimin AV, Delcher AL, Florea L, Kelley DR, Schatz MC, Puiu D, Hanrahan F, Pertea G, Van Tassell CP, Sonstegard TS *et al*: **A whole-genome assembly of the domestic cow, Bos taurus**. *Genome biology* 2009, **10**(4):R42.
29. Canavez FC, Luche DD, Stothard P, Leite KR, Sousa-Canavez JM, Plastow G, Meidanis J, Souza MA, Feijao P, Moore SS *et al*: **Genome sequence and assembly of Bos indicus**. *The Journal of heredity* 2012, **103**(3):342-348.
30. Dobson LK: **Sequencing the Genome of the North American Bison**. *Doctoral dissertation* 2015(Available electronically from http://hdl.handle.net/1969.1/155759).
31. Blanchette M, Kent WJ, Riemer C, Elnitski L, Smit AF, Roskin KM, Baertsch R, Rosenbloom K, Clawson H, Green ED *et al*: **Aligning multiple genomic sequences with the threaded blockset aligner**. *Genome research* 2004, **14**(4):708-715.
32. Kozlov AM, Aberer AJ, Stamatakis A: **ExaML version 3: a tool for phylogenomic analyses on supercomputers**. *Bioinformatics* 2015, **31**(15):2577-2579.
33. Li L, Stoeckert CJ, Jr., Roos DS: **OrthoMCL: identification of ortholog groups for eukaryotic genomes**. *Genome research* 2003, **13**(9):2178-2189.
34. Yates A, Akanni W, Amode MR, Barrell D, Billis K, Carvalho-Silva D, Cummins C, Clapham P, Fitzgerald S, Gil L *et al*: **Ensembl 2016**. *Nucleic acids research* 2016, **44**(D1):D710-716.
35. Hu Q, Ma T, Wang K, Xu T, Liu J, Qiu Q: **The Yak genome database: an integrative database for studying yak biology and high-altitude adaption**. *Bmc Genomics* 2012, **13**:600.
36. Yang Z: **PAML 4: phylogenetic analysis by maximum likelihood**. *Molecular biology and evolution* 2007, **24**(8):1586-1591.

37. Wang, K; Wang, L; Lenstra, J, A; Jian, J; Hu, Q; Yang, Y; Mi, D; Qiu, Q; Ma, T; Abbot, R; Liu, J (2017): **Draft genome of European bison (wisent), *Bison bonasus*.** GigaScience Database. http://dx.doi.org/10.5524/100254

1

2

Table 1. Comparison of genome assembly and gene annotation in wisent, yak and taurine cattle

Species	Assembled genome size (Mb)	Scaffold N50 (Mb)	Contig N50 (kb)	Gene number	Mean gene length (bp)
Wisent	2,575.96	4.69	14.53	21,542	31,458.00
Yak	2,656.77	1.40	20.45	22,282	29,106.90
Taurine Cattle	2,673.16	105.98	88.13	20,526	40,944.10

Additional files

Figure S1: K-mer (k=23) distribution in wisent.

Figure S2: Phylogeny relationship of the assembled mitochondrial sequence.

Figure S3: Sequencing depth of the assembled wisent genome.

Figure S4: FRCurve of six genome assemblies.

Figure S5: Counts of InDels in coding regions, showing an enrichment of multiples of three bases.

Figure S6: Comparison of the composition of repetitive elements in wisent and yak.

Figure S7: Comparison of gene lengths, intron lengths, exon lengths and exon numbers in the genomes of taurine cattle, wisent and yak.

Figure S8: Synteny relationship of wisent and taurine cattle.

Figure S9. Summary of the number of chromosomes that a given scaffold of wisent could be aligned to.

Figure S10. Density of breakpoints (number per million bases) in different regions of genome.

Figure S11: Divergence of American-European bison and taurine–zebu cattle.

Figure S12: Phylogeny relationships within the *Bovini* tribe.

Figure S13: Venn diagram of gene families within five species. Wisent and yak shared the largest number of specific gene families.

Table S1: Summary of sequenced reads.

Table S2: Summary statistics of the genome assembly of wisent.

Table S3: Assembly statistics from published animal genomes generated since 2012.

Table S4: Summary of BUSCO analysis by counting matches to 3023 single-copy orthologs.

Table S5: Summary of CEGMA analysis.

Table S6: The distribution of SNVs in the wisent genome.

Table S7: The distribution of InDels in the wisent genome.

Table S8: Summary statistics of interspersed repeat regions in wisent.

Table S9: Summary statistics of non-coding RNAs in wisent.

Table S10: Summary of breakpoints of wisent and taurine cattle.

Table S11: Summary of synteny alignments.

Table S12: Mean genomic divergence between each species.

Table S13: Summary statistics of gene families in 7 species.

Table S14: Genes subject to positive selection in wisent.

Table S15: Enriched gene ontology of positively selected genes.

Additional file 1

Click here to access/download

Supplementary Material

Wisent Genome- Giga Science-Additional file 1.docx

Personal Cover Click here to download Personal Cover Cover letter.docx

Dear Editor,

Thank you very much for returning our manuscript GIGA-D-16-00043R2 entitled 'The genome sequence of the wisent (*Bison bonasus*)'.

We submit here the revised manuscript and hope that the revised manuscript is more suitable for the publication in Giga Science. If you have any questions, please do not hesitate to contact the corresponding author at any time.

Thank you again for your time and efforts in handling our manuscript.

Best wishes

Sincerely yours,

Jianquan
