# Supplementary material for: The genome sequence of the wisent (Bison bonasus)
Source: Gigascience. 2017 Mar 10;6(4):1–5. doi: 10.1093/gigascience/gix016 (PMC5530314; doi:10.1093/gigascience/gix016)
Supplement: Supplemental material — Figure S1: K-mer (k = 23) distribution in wisent. Figure S2: Phylogeny relationship of the assembled mitochondrial sequence. Figure S3: Sequencing depth of the assembled wisent genome. Figure S4: FRCurve of six genome assemblies. Figure S5: Counts of InDels in coding regions, showing an enrichment of multiples of three bases. Figure S6: Comparison of the composition of repetitive elements in wisent and yak. Figure S7: Comparison of gene lengths, intron lengths, exon lengths and exon numbers in the genomes of taurine cattle, wisent and yak. Figure S8: Synteny relationship of wisent and taurine cattle. Figure S9: Summary of the number of chromosomes that a given scaffold of wisent could be aligned to. Figure S10: Density of breakpoints (number per million bases) in different regions of genome. Figure S11: Divergence of American-European bison and taurine–zebu cattle. Figure S12: Phylogeny relationships within the Bovini tribe. Figure S13: Venn diagram of gene families within five species. Wisent and yak shared the largest number of specific gene families. Table S1: Summary of sequenced reads. Table S2: Summary statistics of the genome assembly of wisent. Table S3: Assembly statistics from published animal genomes generated since 2012. Table S4: Summary of BUSCO analysis by counting matches to 3023 single-copy orthologs. Table S5: Summary of CEGMA analysis. Table S6: The distribution of SNVs in the wisent genome. Table S7: The distribution of InDels in the wisent genome. Table S8: Summary statistics of interspersed repeat regions in wisent. Table S9: Summary statistics of noncoding RNAs in wisent. Table S10: Summary of breakpoints of wisent and taurine cattle. Table S11: Summary of synteny alignments. Table S12: Mean genomic divergence between each species. Table S13: Summary statistics of gene families in seven species. Table S14: Genes subject to positive selection in wisent. Table S15: Enriched gene ontology of positively selected genes. [file gix016_Supp.docx]

**Additional files**


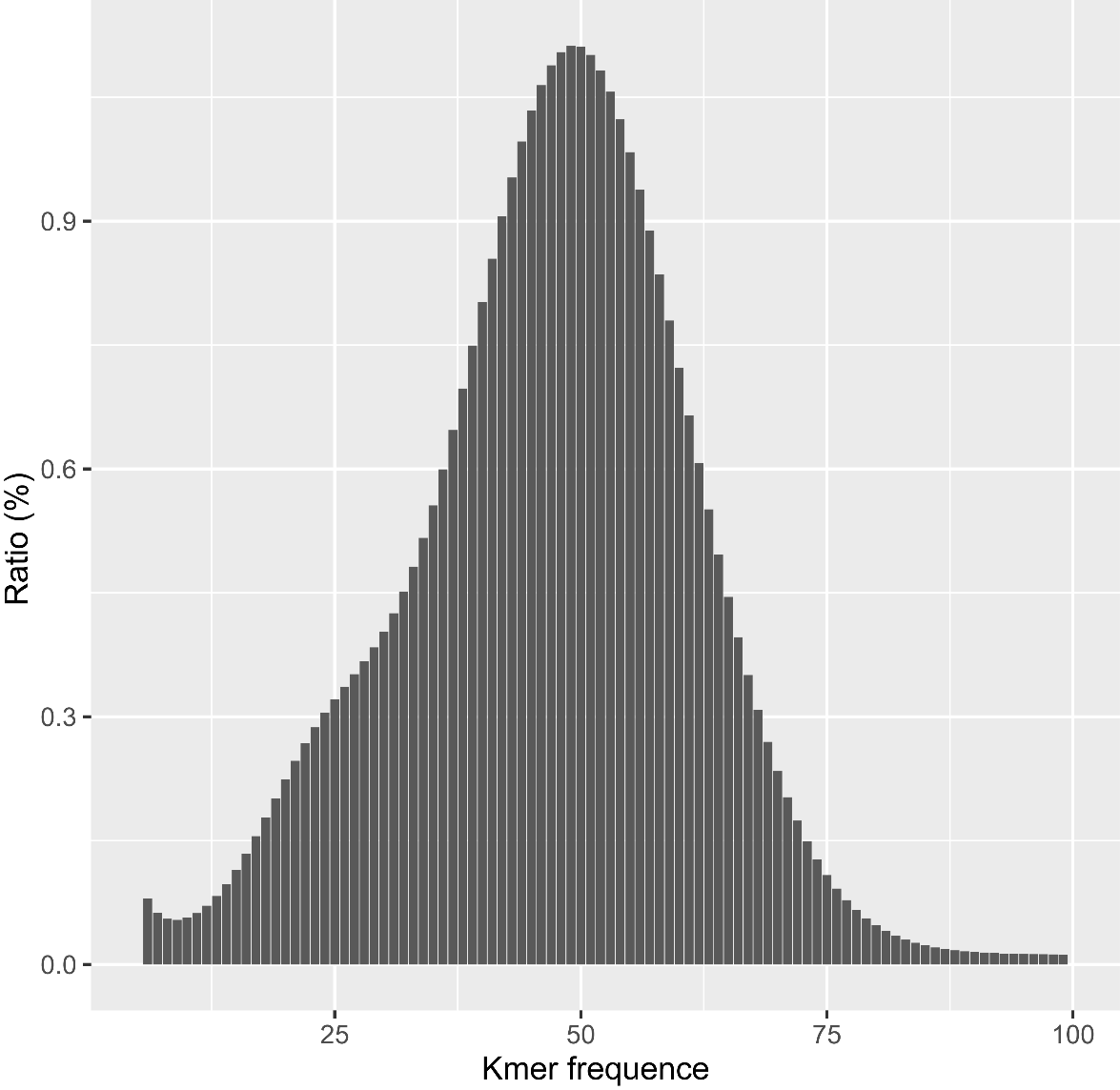


**Figure S1: K-mer (k=23) distribution in wisent.**


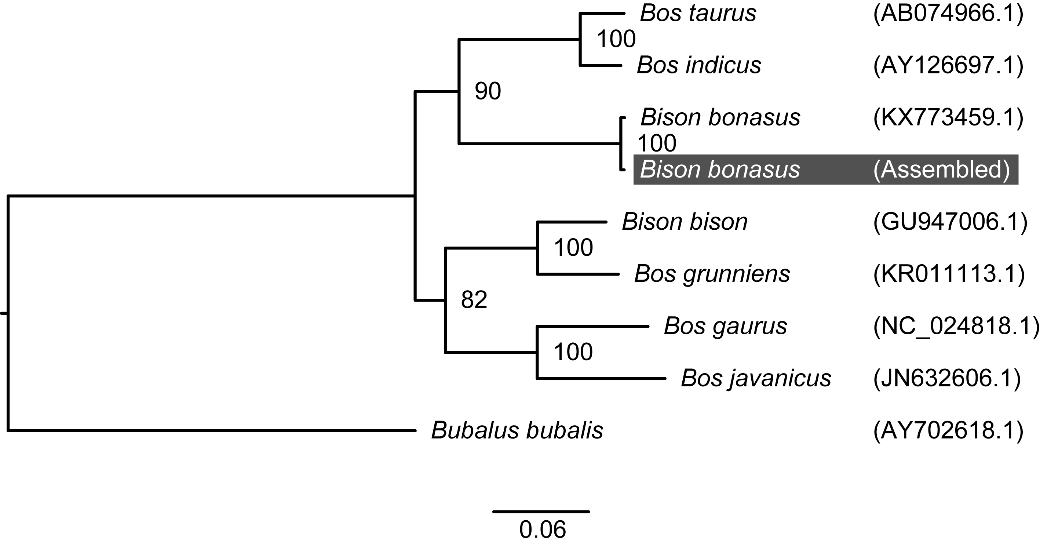


**Figure S2: Phylogeny relationship of the assembled mitochondrial sequence.**


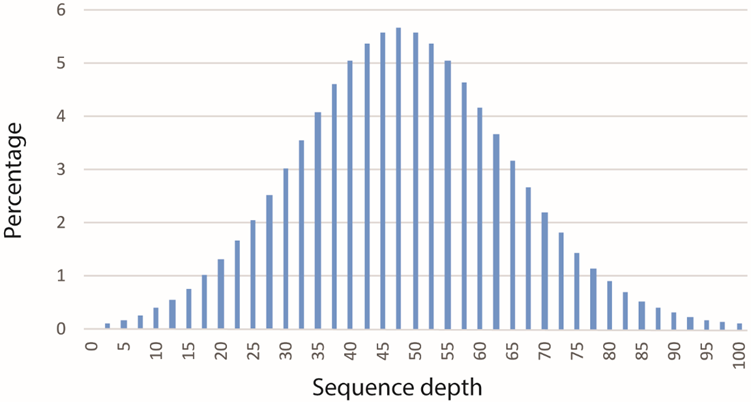


**Figure S3: Sequencing depth of the assembled wisent genome.**


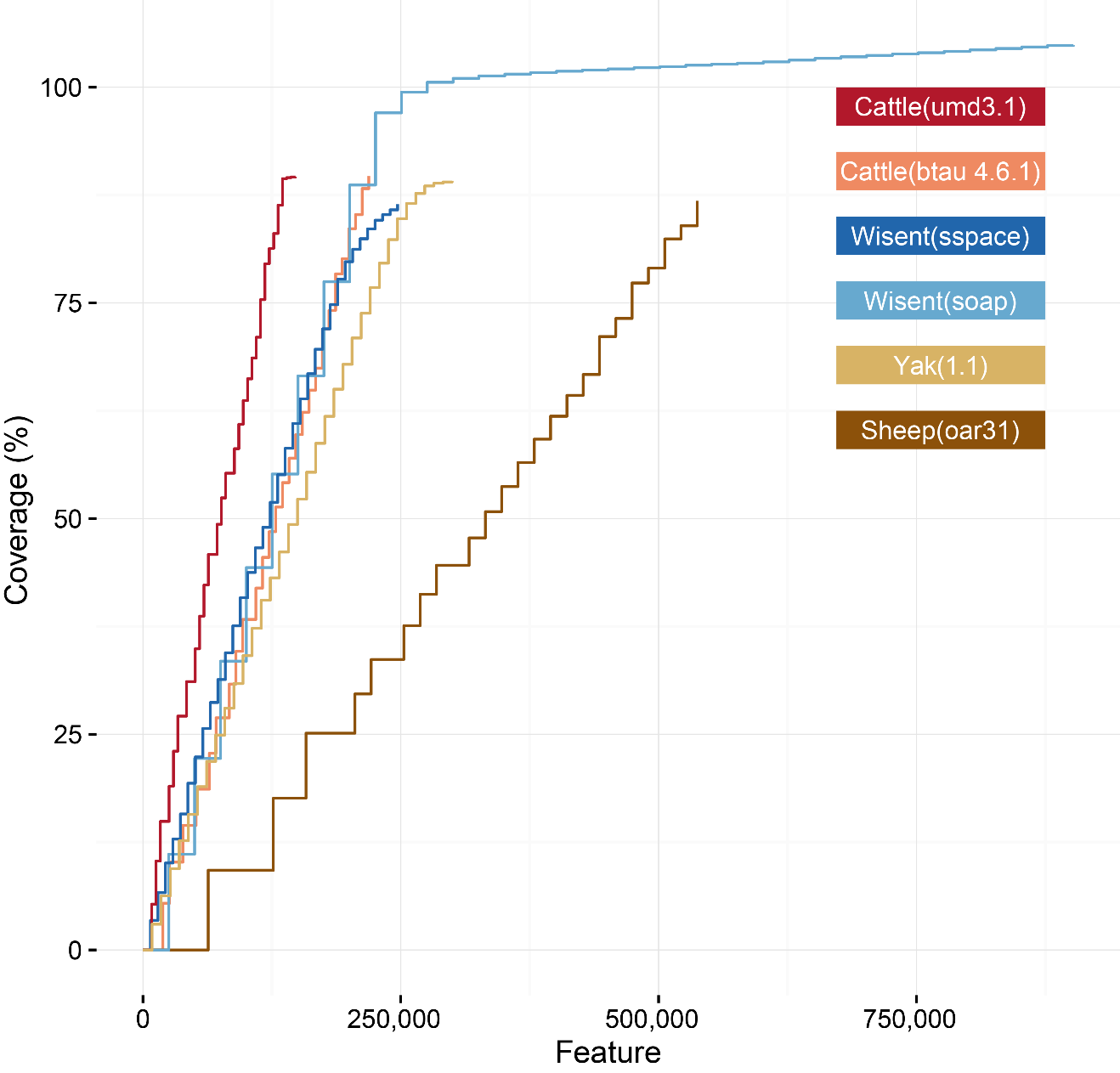


**Figure S4: FRCurve of six genome assemblies.** The FRCurve were calculated by the software FRC_align (<https://github.com/vezzi/FRC_align>) . The two versions of genome assemblies exhibit similar accumulation shapes.

**
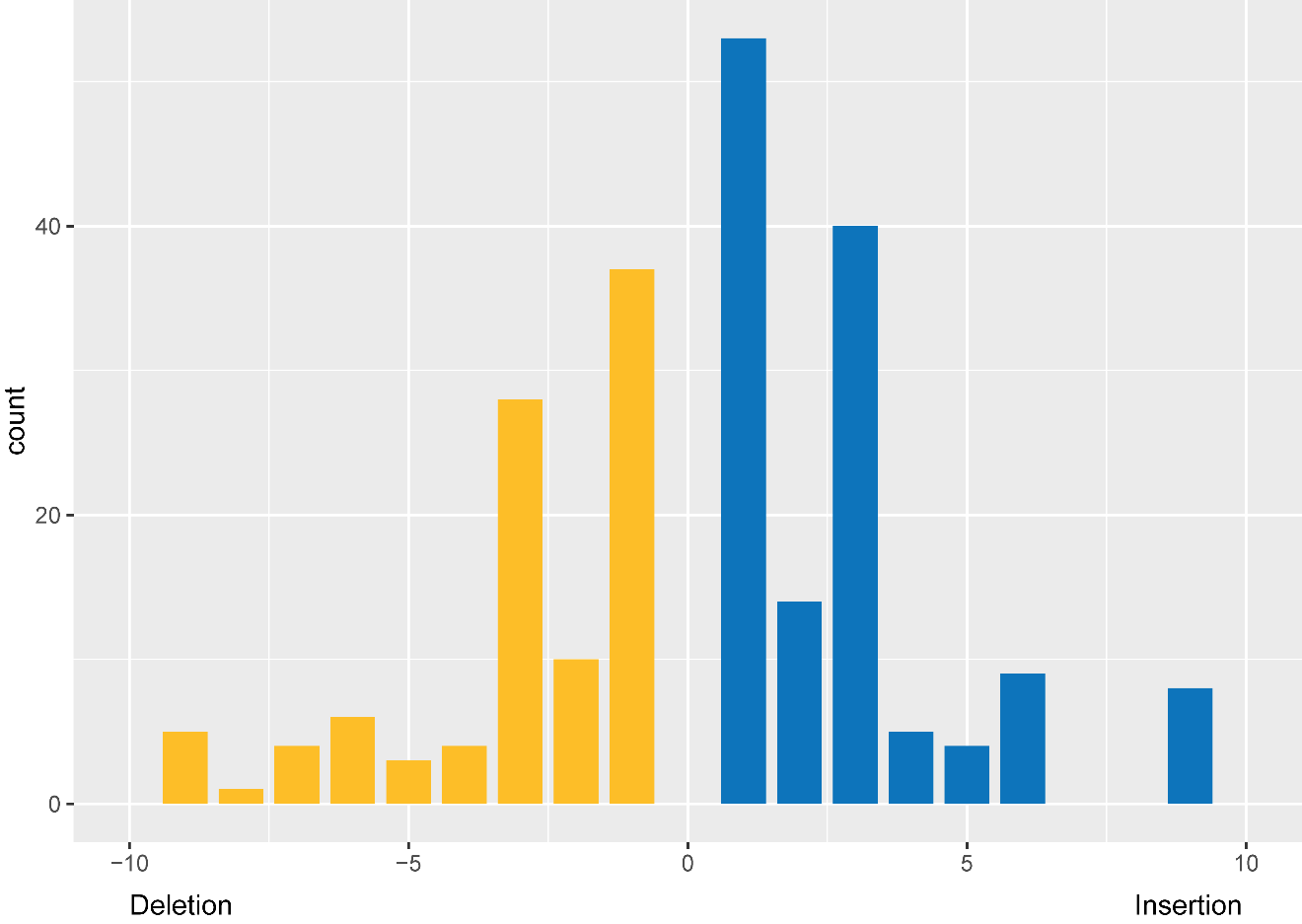
**

**Figure S5: Counts of InDels in coding regions, showing an enrichment of multiples of three bases.**

**Figure S6: Comparison of the composition of repetitive elements in wisent and yak.**


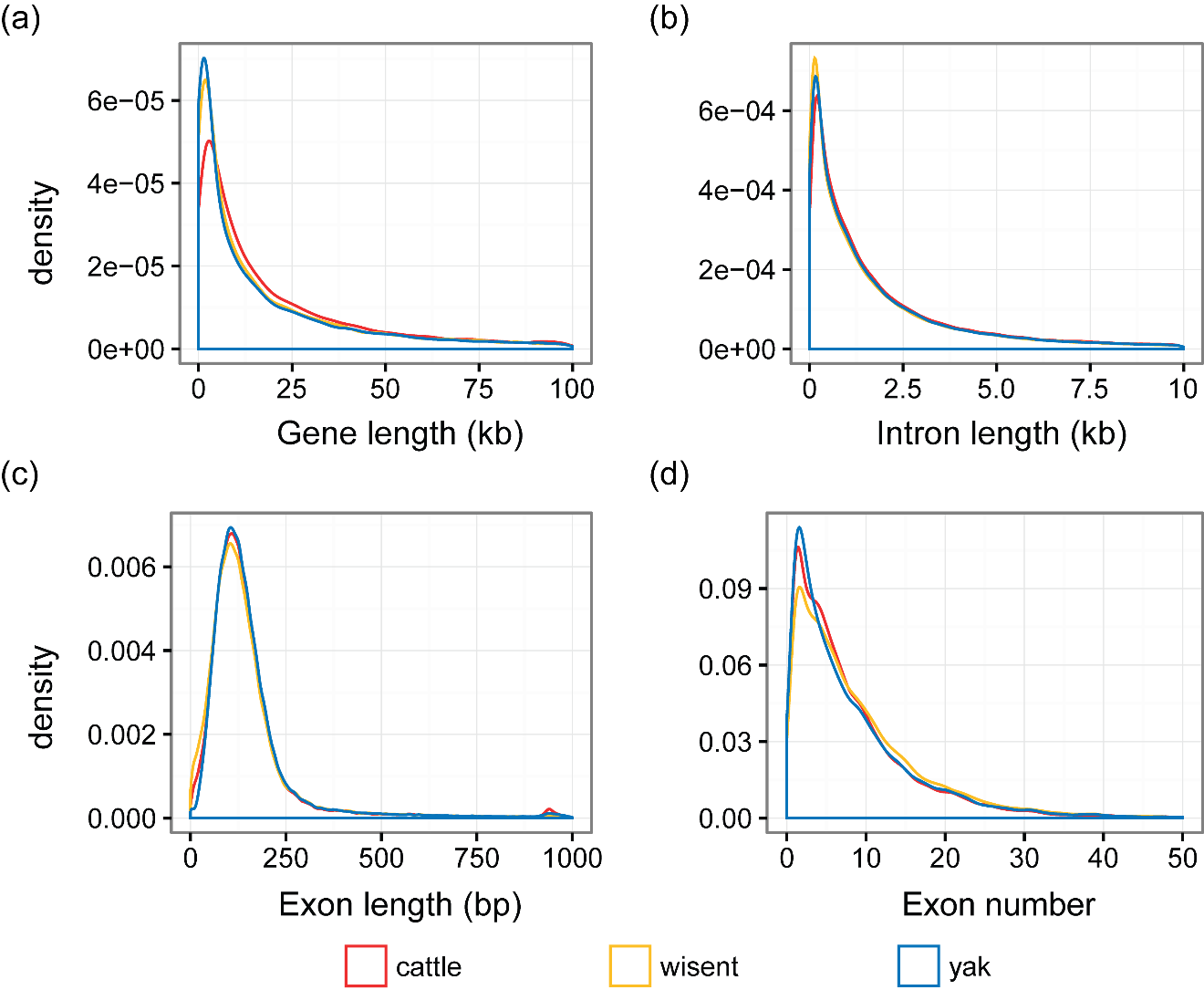


**Figure S7: Comparison of gene lengths, intron lengths, exon lengths and exon numbers in the genomes of taurine cattle, wisent and yak.**

**
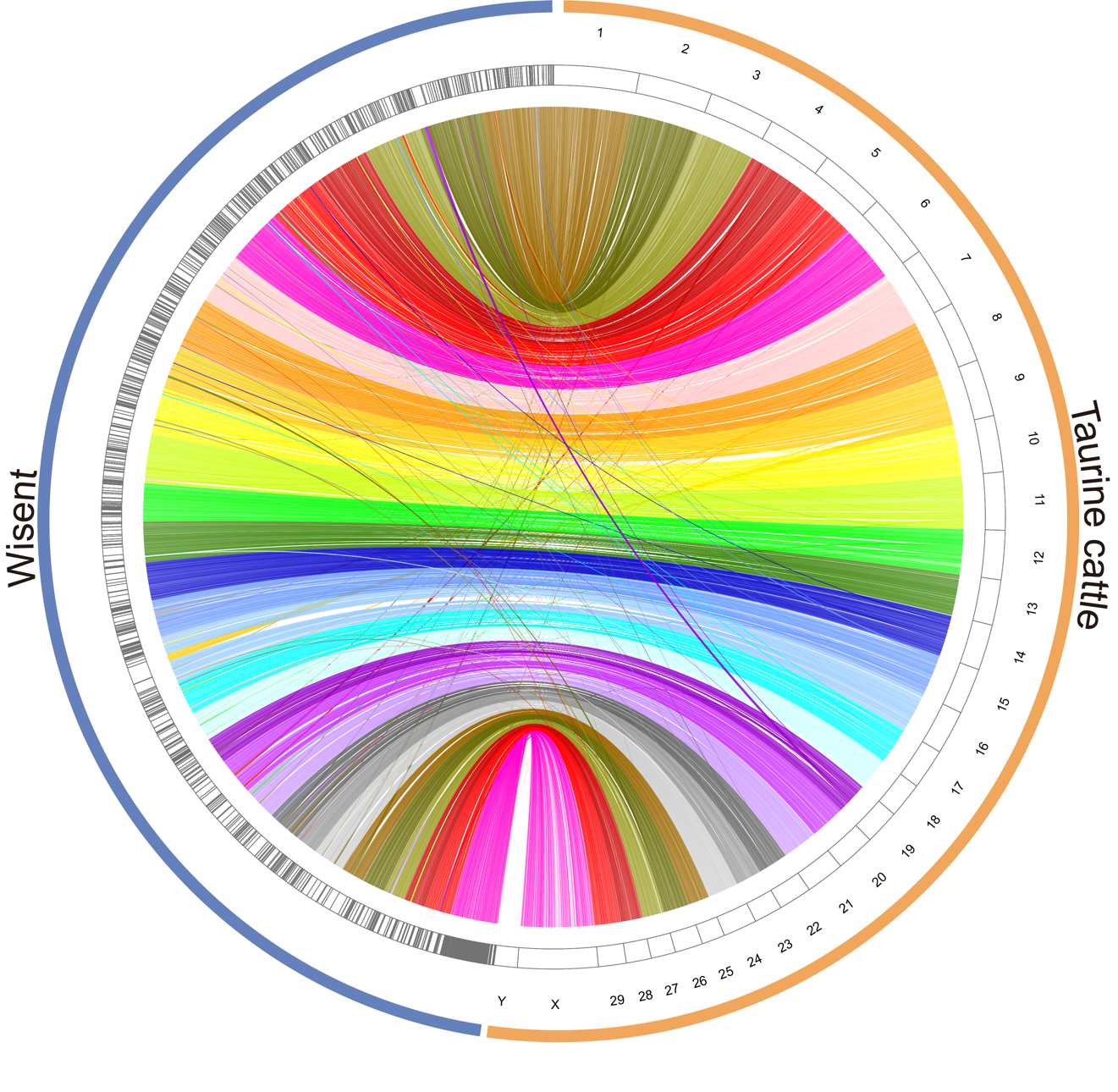
**

**Figure S8: Synteny relationship of wisent and taurine cattle.**


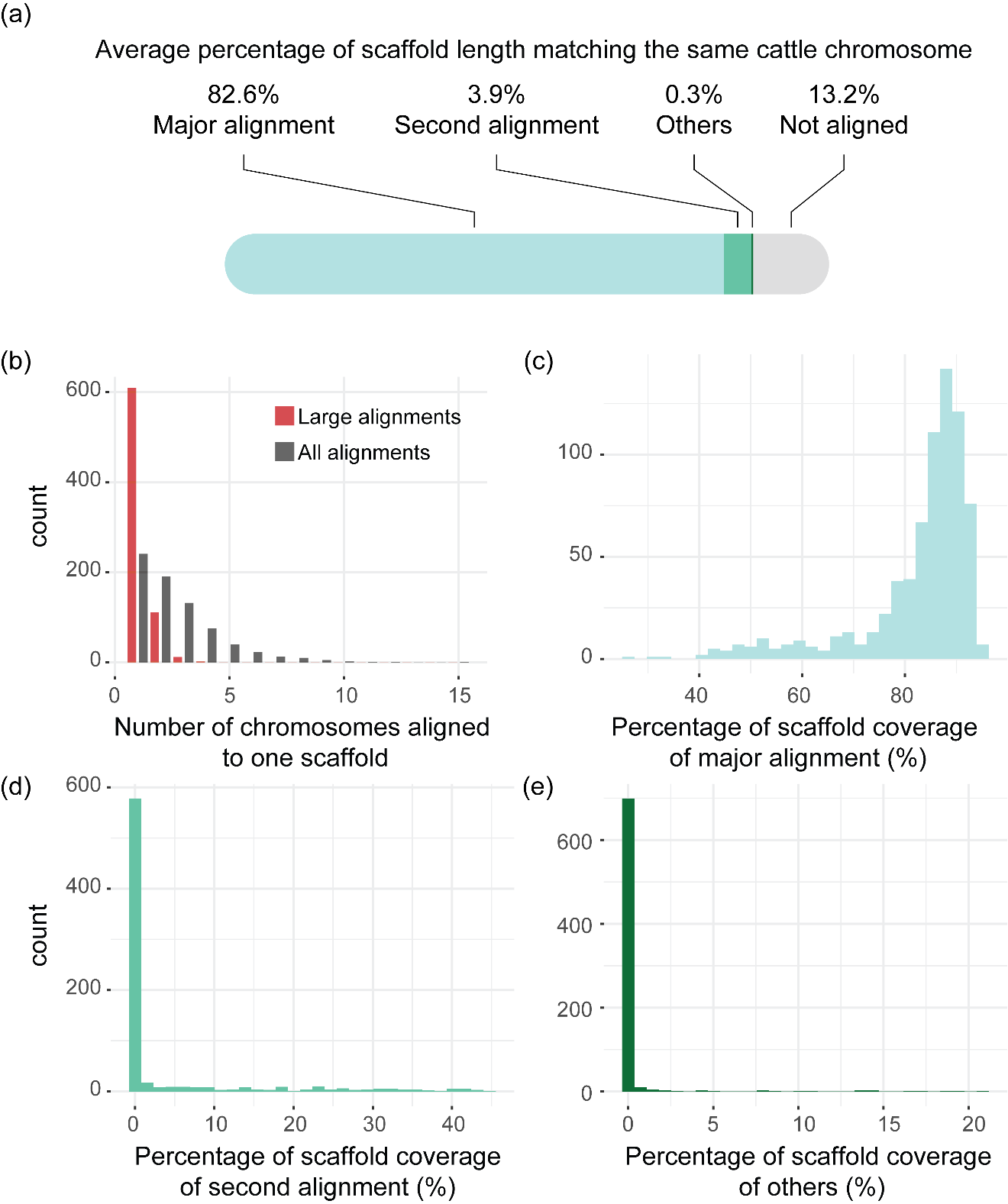
**Figure S9. Summary of the number of chromosomes that a given scaffold of wisent could be aligned to.** (a) The length of alignments from the same chromosome (cattle) were summed and the alignments (sum for one cattle chromosome) with the largest percent of the length of a wisent scaffold (82.6% if averaged over all scaffolds) was named ‘Major alignment’. The alignments (sum of one chromosome of cattle) with second largest percent of the length was named as ‘Second alignment’. The other alignments (from the other chromosomes) was named as ‘Others’. (b) The alignment (from one chromosome) with a percent larger than 5% of a scaffold (not ‘Major alignment’) was named ‘Large alignment’. (c) Histogram of percent of ‘Major alignment’. (d) Histogram of percent of ‘Second alignment’. (e) Histogram of percent of ‘Others’.


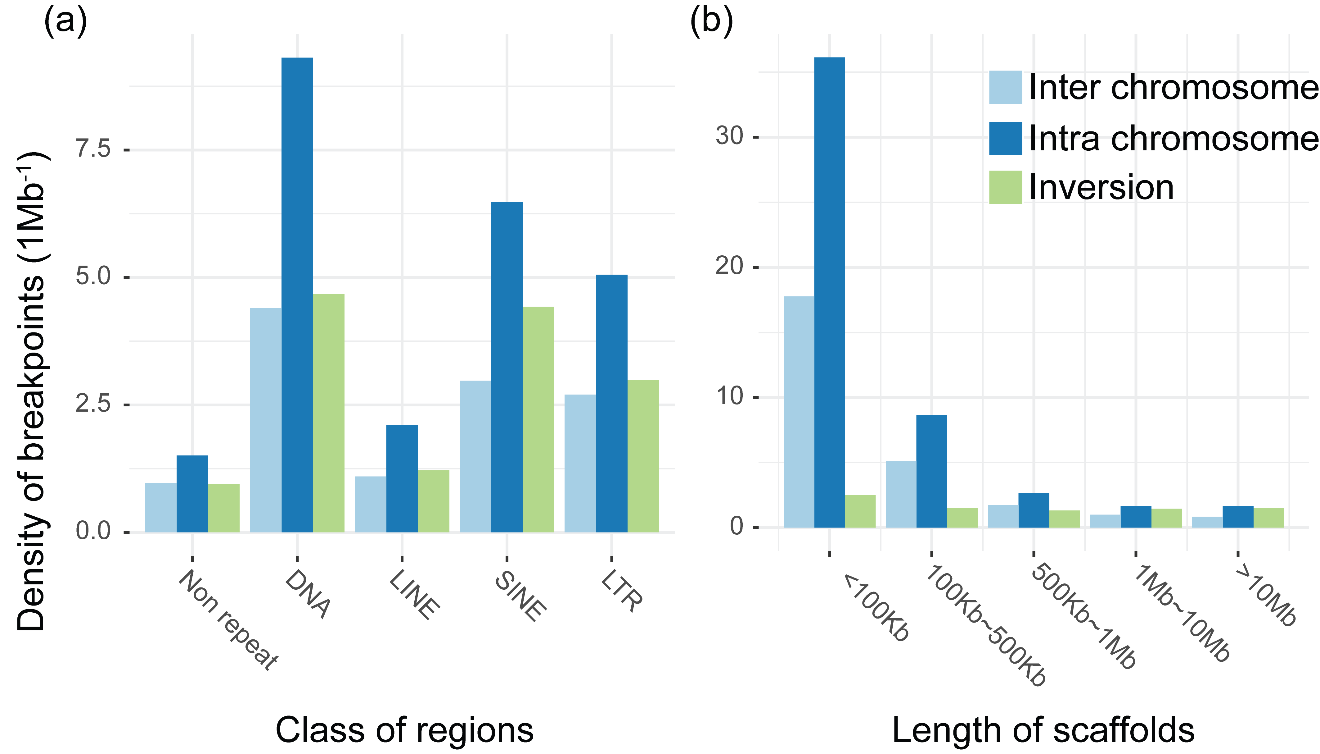


**Figure S10. Density of breakpoints (number per million bases) in different regions of genome.** (a) The breakpoints were counted in non-repeat regions and different types of repetitive regions. (b) The breakpoints were counted in scaffolds with different lengths.


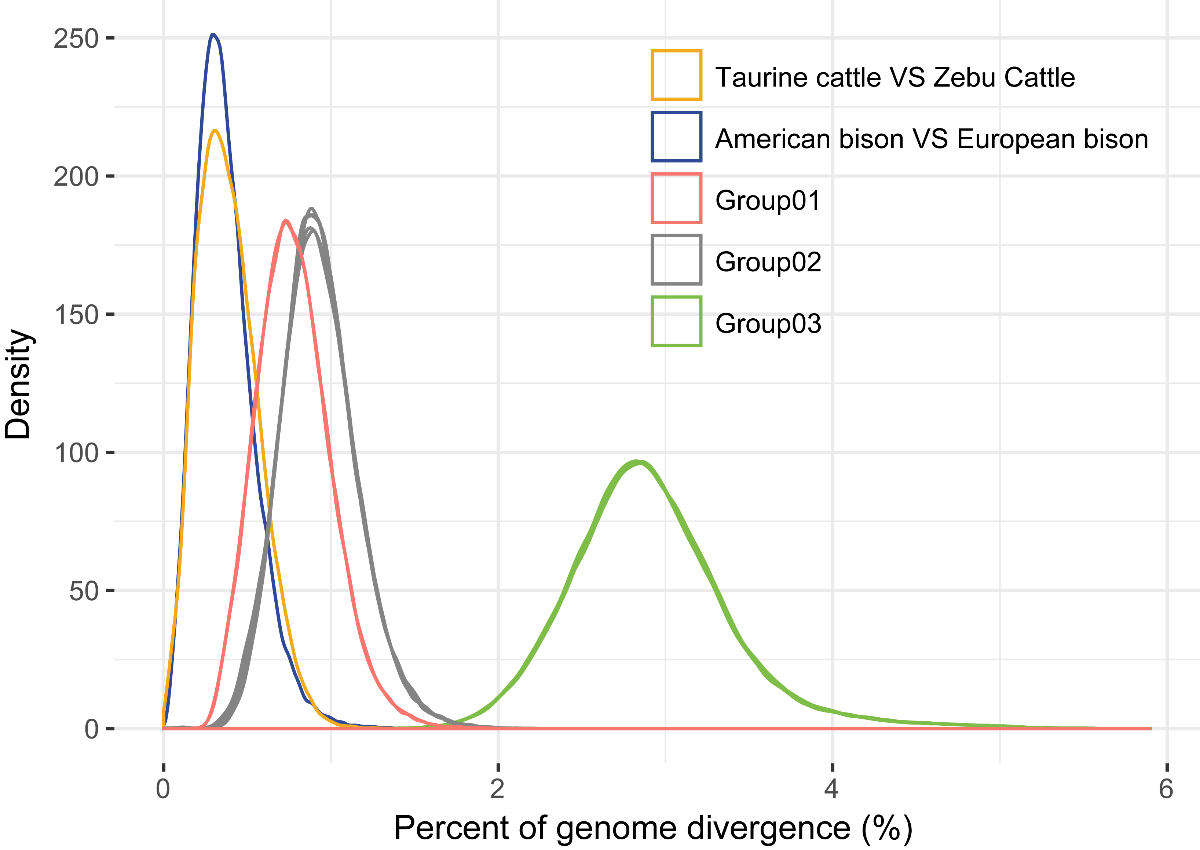


**Figure S11: Divergence of American-European bison and taurine–zebu cattle. Group01**: American bison or wisent vs yak; **Group02**: American bison, wisent or yak vs taurine cattle or zebu; **Group03**: water buffalo vs the other species.


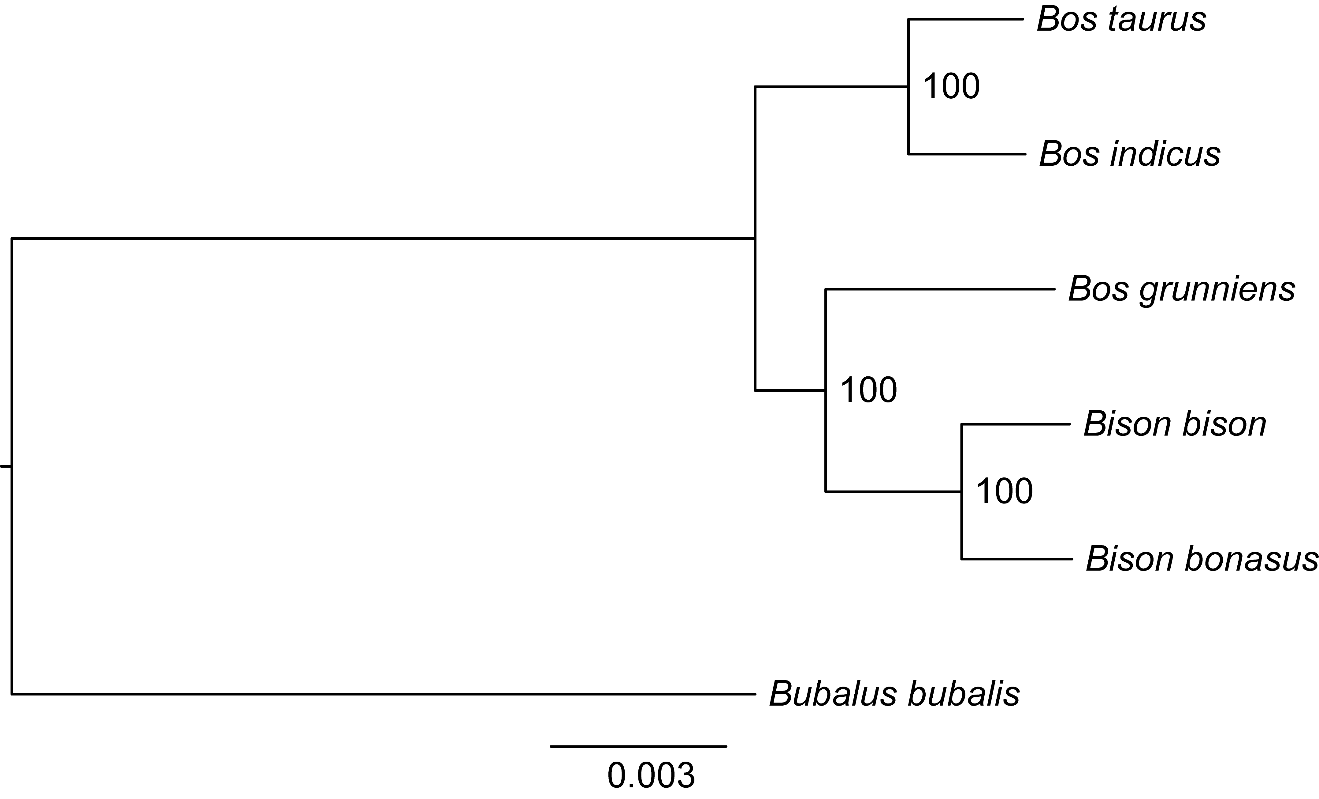


**Figure S12: Phylogeny relationships within the *Bovini* tribe.**

**
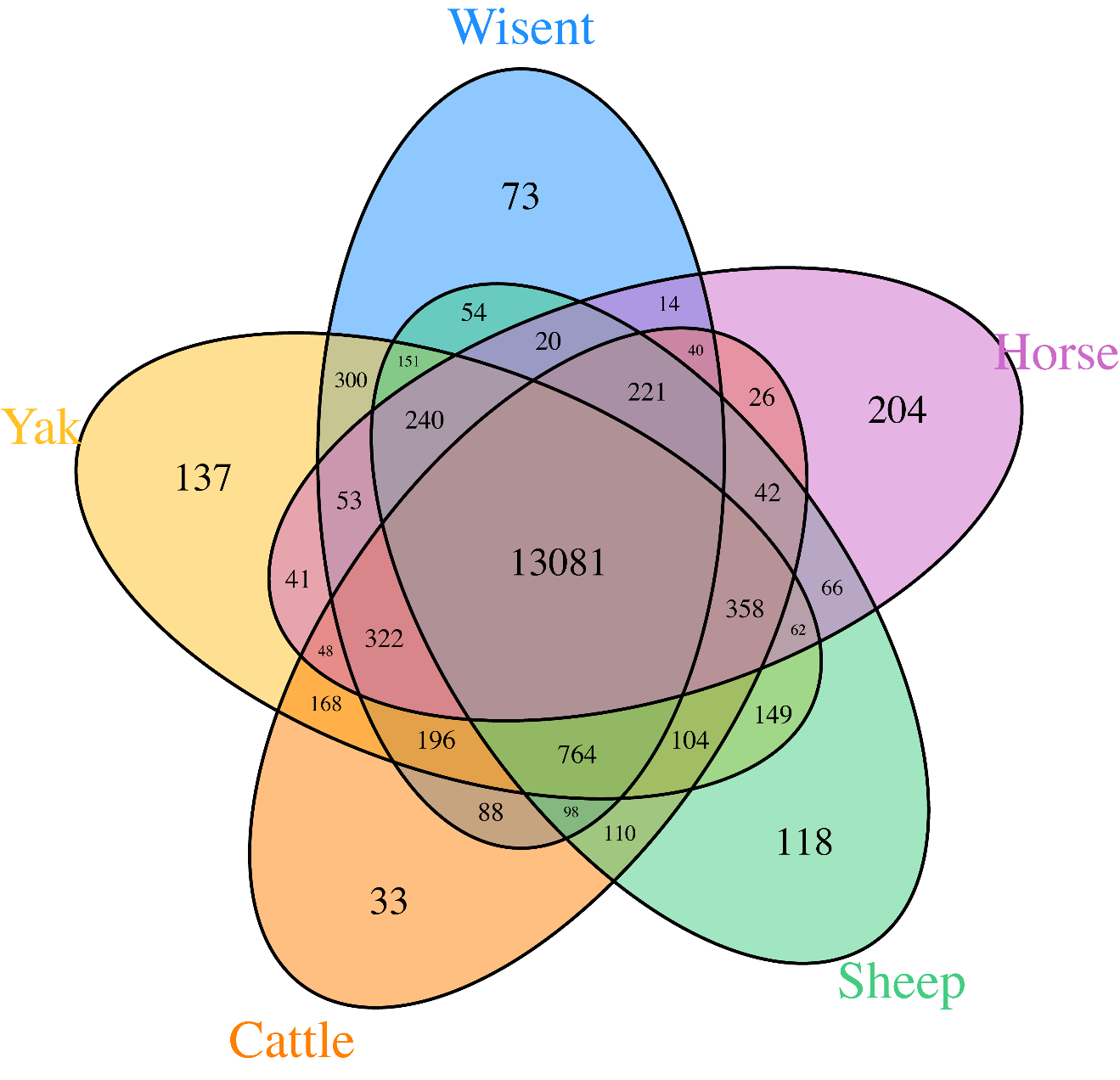
**

**Figure S13: Venn diagram of gene families within five species.** Wisent and yak shared the largest number of specific gene families.

**Table S1: Summary of sequenced reads.**

| **Insert Size (bp)** | **Type** | **Bases sequenced (Gb)** | **Reads Length (bp)** | **Sequence coverage (×)** | **Physical coverage (×)** | **Deposit number** |
| --- | --- | --- | --- | --- | --- | --- |
| 170 | Paired-end | 28.64 | 100 | 9.54 | 8.11 | SRR3530551 |
| 200 | Paired-end | 75.57 | 100 | 25.19 | 25.19 | SRR3530563 |
| 500 | Paired-end | 59.21 | 100 | 19.74 | 49.34 | SRR3530566 |
| 800 | Paired-end | 57.03 | 100 | 19.01 | 76.04 | SRR3530578 |
| 2,000 | Mate-paired | 47.16 | 49 | 15.72 | 320.82 | SRR3531966 |
| 5,000 | Mate-paired | 39.88 | 49 | 13.29 | 678.23 | SRR3531969 |
| 10,000 | Mate-paired | 29.92 | 49 | 9.97 | 1,017.69 | SRR3531971 |
| 20,000 | Mate-paired | 29.06 | 49 | 9.69 | 1,976.87 | SRR3531973 |
| Total |  | 366.47 |  | 122.15 | 4,152.29 |  |

**Table S2: Summary statistics of the genome assembly of wisent.**

|  | **Contig** | | **Scaffold** | |
| --- | --- | --- | --- | --- |
|  | **Size(kb)** | **Number** | **Size(kb)** | **Number** |
| N90 | 3.87 | 171,963 | 628.59 | 677 |
| N80 | 6.42 | 123,531 | 1,700.71 | 436 |
| N70 | 8.9 | 91,229 | 2,571.92 | 313 |
| N60 | 11.55 | 67,069 | 3,570.06 | 229 |
| N50 | 14.53 | 48,119 | 4,691.65 | 167 |
| Longest | 236,229 | | 31,654,992 | |
| Total Number | 29,074 | | 339,090 | |
| Total Size | 2,450,794,037 | | 2,575,955,350 | |

**Table S3: Assembly statistics from published animal genomes generated since 2012.**

| **Species Name** | **Latin Name** | **Publish date** | **Sequencing platform** | **Assembled genome size (Gb)** | **scaffold** | | | | **contig** | | | | |
| --- | --- | --- | --- | --- | --- | --- | --- | --- | --- | --- | --- | --- | --- |
|  |  |  |  |  | **total number** | **N50 (Mb)** | **N50 number** | **largest (Mb)** | **total number** | **N50 (Kb)** | **N50 number** | **largest (Kb)** |  |
| Bactrian camel [1] | *Camelus bactrianus* | 2012 | Illumina & SOLiD | 2.38 | 120,352 | 2.00 |  |  |  | 85.30 |  |  |  |
| Duroc pig [2] | *Sus scrofa* | 2012 | Illumina & Sanger | 2.60 |  | 6.37 |  |  |  | 80.70 |  | 1,600.00 |  |
| Wuzhishan Pig [3] | *Sus scrofa* | 2012 | Illumina | 2.60 |  | 5.40 |  | 21.41 |  | 23.50 |  | 230.00 |  |
| Gorilla [4] | *Gorilla gorilla gorilla* | 2012 | Pacbio | 3.04 | 22,164 | 0.91 |  | 10.25 | 465,847 | 11.80 |  | 191.56 |  |
| Baiji dolphin [5] | *Lipotes vexillifer* | 2013 | Illumina | 2.80 | 1,149,419 | 2.26 | 328 | 11.54 | 1,274,530 | 30.00 | 24,909 | 329.00 |  |
| Mink whale [6] | *Balaenoptera acutorostrata* | 2013 | Illumina | 2.76 | 104,325 | 12.80 | 57 | 51.45 | 278,792 | 22.60 | 31,010 | 178.00 |  |
| Goat [7] | *Capra hircus* | 2013 | Illumina | 2.66 | 285,383 | 3.06 | 254 | 16.33 | 542,145 | 18.70 | 39,408 |  |  |
| Tibetan antelope [8] | *Pantholops hodgsonii* | 2013 | Illumina | 2.70 | 15,996 | 2.76 | 296 | 13.45 | 299,021 | 18.60 | 39,469 | 249.00 |  |
| Tibetan wild boar [9] | *Sus scrofa* | 2013 | Illumina | 2.43 |  | 1.05 | 714 | 6.12 |  | 20.40 | 32,634 | 278.36 |  |
| Chinese hamster [10] | *Cricetulus griseus* | 2013 | Illumina | 2.60 | 286,619 | 2.49 | 271 | 10.80 | 458,620 | 26.00 | 26,456 | 219.44 |  |
| Srandt's bat [11] | *Myotis brandtii* | 2013 | Illumina | 2.00 | 677,946 | 3.11 | 192 | 22.12 | 844,285 | 21.10 | 27,229 | 217.90 |  |
| Tree shrew [12] | *Tupaia belangeri* | 2013 | Illumina | 3.20 |  | 3.66 |  | 19.27 |  | 22.00 |  | 188.00 |  |
| Sheep [13] | *Ovis aries* | 2014 | Illumina | 2.60 |  | 2.20 |  |  |  | 40.00 |  |  |  |
| Rabbit [14] | *Oryctolagus cuniculus* | 2014 | Illumina | 2.66 |  | 35.90 |  |  |  | 64.70 |  |  |  |
| Ferret [15] | *Mustela putorius furo* | 2014 | Illumina | 2.41 |  | 9.30 |  |  |  | 44.80 |  |  |  |
| Blind subterranean Mole Rat [16] | *Spalax galili* | 2014 | Illumina | 3.06 | 162,113 | 3.60 | 238 | 15.90 | 378,056 | 27.58 | 31,146 | 285.52 |  |
| Snub-nosed monkeys [17] | *Rhinopithecus roxellana* | 2014 | Illumina | 3.05 | 135,657 | 1.55 | 533 | 9.85 | 330,506 | 25.50 | 32,936 | 280.83 |  |
| Marmoset [18] | *Callithrix jacchus* | 2014 | Sanger | 2.26 | 16,089 | 6.70 | 113 | 39.89 | 202,484 | 29.00 | 27,104 | 325.72 |  |
| white-cheeked gibbon [19] | *Nomascus leucogenys* | 2014 | Sanger | 2.93 | 17,976 | 22.69 |  |  | 197,908 | 35.15 |  |  |  |
| Bowhead whale [20] | *Balaena mysticetus* | 2015 | Illumina | 2.87 |  | 0.88 | 7,227 |  |  | 34.80 | 113,673 |  |  |
| Donkey [21] | *Equus asinus* | 2015 | Illumina & 454 | 2.36 | 510,501 | 3.80 | 6,145 | 1.09 | 1,262,621 | 66.70 | 99,025 | 67.00 |  |
| Blue-Eyed Black Lemur [22] | *Eulemur ﬂavifrons* | 2015 | Illumina | 2.68 | 21,210 | 0.42 |  | 3.30 | 280,211 | 16.30 |  | 222.30 |  |
| Arabian camel [23] | *Camelus dromedarius* | 2016 | Illumina | 2.06 | 140,480 | 8.76 | 69 | 46.54 | 282,890 | 24.00 | 23,893 | 203.00 |  |
| Giraffa [24] | *Giraffa camelopardalis* | 2016 | Illumina | 2.90 | 2,051,673 | 0.34 | 2,088 | 5.62 | 2,133,680 | 47.20 | 16,327 | 540.05 |  |
| Okapi [24] | *Okapia johnstoni* | 2016 | Illumina | 3.30 | 3,721,563 | 0.10 | 6,933 | 2.17 | 3,808,993 | 33.70 | 22,986 | 592.60 |  |
| Gorilla [25] | *Gorilla gorilla gorilla* | 2016 | SMRT | 3.08 | 554 | 23.10 |  | 110.02 | 16,073 | 9,560.00 |  | 36,220.00 |  |

**Table S4: Summary of BUSCO analysis by counting matches to 3023 single-copy orthologs.**

| **BUSCO mode** | **Species** | **Complete one-to-one match to ortholog** | **Complete match of multi gene copies to ortholog** | **Fragmented match to ortholog** | **Total number of matches to ortholog** | **No match to ortholog** |
| --- | --- | --- | --- | --- | --- | --- |
| Genome | Wisent | 2522 (83.4%) | 55 | 292 | 2,869 (94.9%) | 154 |
|  | Yak | 2438 (80.6%) | 41 | 375 | 2,854 (94.4%) | 169 |
|  | Taurine cattle (Btau 4.6.1) | 2321 (76.4%) | 59 | 385 | 2,765 (91.5%) | 258 |
|  | Taurine cattle  (umd 3.1) | 2416 (79.9%) | 55 | 388 | 2,859 (94.6%) | 164 |
| OGS | Wisent | 2846 (94.1%) | 78 | 67 | 2,992 (99.0%) | 32 |
|  | Yak | 2881 (95.3%) | 58 | 54 | 2,993 (99.0%) | 30 |
|  | Taurine cattle (Btau 4.6.1) | 2530 (83.7%) | 47 | 89 | 3,006 (88.2%) | 357 |
|  | Taurine cattle  (umd 3.1) | 2728 (90.2%) | 229 | 49 | 3,006 (99.4%) | 17 |

**Table S5: Summary of CEGMA analysis.**

| **Species** |  | **#Proteins ^1^** | **Completeness (%) ^2^** | **#Total ^3^** | **Average ^4^** | **Ortho (%) ^5^** |
| --- | --- | --- | --- | --- | --- | --- |
| Wisent | Complete | 169 | 68.15 | 285 | 1.69 | 42.6 |
|  | Group 1 | 40 | 60.61 | 58 | 1.45 | 27.5 |
|  | Group 2 | 36 | 64.29 | 51 | 1.42 | 30.56 |
|  | Group 3 | 41 | 67.21 | 70 | 1.71 | 46.34 |
|  | Group 4 | 52 | 80 | 106 | 2.04 | 59.62 |
|  |  |  |  |  |  |  |
|  | Partial | 234 | 94.35 | 552 | 2.36 | 68.38 |
|  | Group 1 | 59 | 89.39 | 128 | 2.17 | 61.02 |
|  | Group 2 | 53 | 94.64 | 106 | 2 | 64.15 |
|  | Group 3 | 59 | 96.72 | 142 | 2.41 | 71.19 |
|  | Group 4 | 63 | 96.92 | 176 | 2.79 | 76.19 |
| Taurine cattle (Btau4.6.1) | Complete | 149 | 60.08 | 248 | 1.66 | 38.93 |
|  | Group 1 | 34 | 51.52 | 50 | 1.47 | 29.41 |
|  | Group 2 | 34 | 60.71 | 48 | 1.41 | 29.41 |
|  | Group 3 | 35 | 57.38 | 59 | 1.69 | 42.86 |
|  | Group 4 | 46 | 70.77 | 91 | 1.98 | 50 |
|  | Partial | 225 | 90.73 | 481 | 2.14 | 63.11 |
|  | Group 1 | 56 | 84.85 | 104 | 1.86 | 48.21 |
|  | Group 2 | 53 | 94.64 | 98 | 1.85 | 56.6 |
|  | Group 3 | 56 | 91.8 | 122 | 2.18 | 67.86 |
|  | Group 4 | 60 | 92.31 | 157 | 2.62 | 78.33 |
|  |  |  |  |  |  |  |
| Taurine cattle (UMD3.1) | Complete | 153 | 61.69 | 252 | 1.65 | 37.91 |
|  | Group 1 | 36 | 54.55 | 51 | 1.42 | 25 |
|  | Group 2 | 34 | 60.71 | 49 | 1.44 | 29.41 |
|  | Group 3 | 36 | 59.02 | 60 | 1.67 | 38.89 |
|  | Group 4 | 47 | 72.31 | 92 | 1.96 | 53.19 |
|  |  |  |  |  |  |  |
|  | Partial | 231 | 93.15 | 520 | 2.25 | 67.1 |
|  | Group 1 | 57 | 86.36 | 116 | 2.04 | 59.65 |
|  | Group 2 | 52 | 92.86 | 105 | 2.02 | 59.62 |
|  | Group 3 | 60 | 98.36 | 138 | 2.3 | 71.67 |
|  | Group 4 | 62 | 95.38 | 161 | 2.6 | 75.81 |
|  |  |  |  |  |  |  |
| Yak | Complete | 158 | 63.71 | 257 | 1.63 | 35.44 |
|  | Group 1 | 39 | 59.09 | 55 | 1.41 | 25.64 |
|  | Group 2 | 36 | 64.29 | 50 | 1.39 | 25 |
|  | Group 3 | 33 | 54.1 | 56 | 1.7 | 45.45 |
|  | Group 4 | 50 | 76.92 | 96 | 1.92 | 44 |
|  |  |  |  |  |  |  |
|  | Partial | 236 | 95.16 | 524 | 2.22 | 64.41 |
|  | Group 1 | 59 | 89.39 | 111 | 1.88 | 50.85 |
|  | Group 2 | 53 | 94.64 | 102 | 1.92 | 60.38 |
|  | Group 3 | 61 | 100 | 139 | 2.28 | 67.21 |
|  | Group 4 | 63 | 96.92 | 172 | 2.73 | 77.78 |

^1^ number of 248 ultra-conserved core eukaryotic genes (CEGs) present in genome;

^2^ percentage of 248 ultra-conserved CEGs present;

^3^ total number of CEGs present including putative orthologs;

^4^ average number of orthologs per CEG;

^5^ percentage of detected CEGS having more than 1 ortholog.

**Table S6: The distribution of SNVs in the wisent genome.**

| **Location** | **Count** |
| --- | --- |
| Inter-genetic | 1,468,509 |
| Intron | 450,479 |
| synonymous variant | 10,716 |
| missense variant |  |
| missense | 9,396 |
| stop gain | 129 |
| stop loss | 33 |
| start loss | 38 |
| **Total** | **1,939,300** |

**Table S7: The distribution of InDels in the wisent genome.**

| **Location** | **Count** |
| --- | --- |
| Intergenetic | 116,777 |
| Intron | 38,961 |
| Exonic |  |
| Frameshift | 162 |
| Inframe | 75 |
| **Total** | **155,975** |

**Table S8: Summary statistics of interspersed repeat regions in wisent.**

|  | **Repbase TEs** | | **TE proteins** | | ***De novo*** | | **Combined TEs** | |
| --- | --- | --- | --- | --- | --- | --- | --- | --- |
| **Type** | **Length (bp)** | **% in genome** | **Length (bp)** | **% in genome** | **Length (bp)** | **% in genome** | **Length (bp)** | **% in genome** |
| DNA | 59,617,405 | 2.31 | 6,332,569 | 0.25 | 3,368,273 | 0.13 | 62,017,441 | 2.41 |
| LINE | 585,473,854 | 22.73 | 363,444,610 | 14.11 | 835,954,726 | 32.45 | 1,026,331,447 | 39.84 |
| SINE | 256,023,483 | 9.94 | 0 | 0.00 | 20,269,926 | 0.79 | 268,268,335 | 10.41 |
| LTR | 128,856,647 | 5.00 | 10,421,287 | 0.40 | 361,274,696 | 14.02 | 456,281,995 | 17.71 |
| Other | 1,619 | 0.00 | 0 | 0.00 | 0 | 0.00 | 1,619 | 0.00 |
| Unknown | 0 | 0.00 | 0 | 0.00 | 46,056,648 | 1.79 | 46,056,648 | 1.79 |
| Total | 1,007,184,959 | 39.10 | 380,122,834 | 14.76 | 1,030,198,518 | 39.99 | 1,211,514,182 | 47.03 |

**Table S9: Summary statistics of non-coding RNAs in wisent.**

| **Type** | | **Copy number** | **Average length(bp)** | **Total length(bp)** | **% of genome** |
| --- | --- | --- | --- | --- | --- |
| miRNA |  | 20,613 | 100.44 | 2,070,373 | 8.04% |
| tRNA |  | 35,445 | 72.84 | 2,581,766 | 10.02% |
| rRNA |  | 767 | 99.77 | 76,523 | 0.30% |
|  | 18S | 95 | 122.84 | 11,670 | 0.05% |
|  | 28S | 210 | 148.10 | 31,101 | 0.12% |
|  | 5.8S | 7 | 102.14 | 715 | 0.00% |
|  | 5S | 455 | 72.61 | 33,037 | 0.13% |
| snRNA |  | 1,560 | 114.72 | 178,963 | 0.69% |
|  | CD-box | 308 | 93.11 | 28,678 | 0.11% |
|  | HACA-box | 283 | 135.92 | 38,466 | 0.15% |
|  | splicing | 932 | 115.25 | 107,413 | 0.42% |

**Table S10: Summary of breakpoints of wisent and taurine cattle.**

| **Type of breakpoints** | **Number** |
| --- | --- |
| Inter-chromosomal | 3,554 |
| Intra-chromosomal | 6,270 |
| Inversion | 3,671 |
| **Total** | **13,495** |

**Table S11: Summary of synteny alignments.**

| **Species** | **Length of query^a^** | **Length of reference** | **Length after MultiZ^b^** |
| --- | --- | --- | --- |
| European bison | 2,220,763,552 | 2,221,155,477 | 1,943,195,550 |
| American bison | 2,468,756,559 | 2,469,179,499 | 1,943,199,537 |
| Yak | 2,383,320,043 | 2,383,692,220 | 1,943,240,320 |
| Water buffalo | 2,379,701,246 | 2,378,959,637 | 1,944,238,370 |
| Zebu cattle | 2,381,050,070 | 2,380,835,605 | 1,943,650,242 |
| Taurine cattle | NA | NA | 1,943,498,518 |

^a^ The genome sequences of each species was aligned to the cattle genome. Length of query refers to the alignment length of one-to-one alignment in query sequences. Length of reference refers to the alignment length of one-to-one alignment in reference sequences.

^b^ The alignments were integrated with MultiZ. Length after MultiZ refers to the length of each genome sequence in the alignments.

**Table S12: Mean genomic divergence between each species.**

| **Combination** | **Total site** | **Diff site** | **Percent** |
| --- | --- | --- | --- |
| Water buffalo VS Yak | 1,924,882,809 | 56,048,787 | 2.91% |
| American bison VS Water buffalo | 1,924,882,809 | 56,029,176 | 2.91% |
| Water buffalo VS European bison | 1,924,882,809 | 56,009,658 | 2.91% |
| Water buffalo VS Zebu cattle^a^ | 1,924,882,809 | 55,814,629 | 2.90% |
| Water buffalo VS Taurine cattle | 1,924,882,809 | 55,754,672 | 2.90% |
| American bison VS Zebu cattle | 1,924,882,809 | 18,006,742 | 0.94% |
| Zebu cattle VS European bison | 1,924,882,809 | 18,005,107 | 0.94% |
| Zebu cattle VS Yak | 1,924,882,809 | 17,973,351 | 0.93% |
| Taurine cattle VS European bison | 1,924,882,809 | 17,944,323 | 0.93% |
| American bison VS Taurine cattle | 1,924,882,809 | 17,944,102 | 0.93% |
| Taurine cattle VS Yak | 1,924,882,809 | 17,863,913 | 0.93% |
| European bison VS Yak | 1,924,882,809 | 15,050,489 | 0.78% |
| American bison VS Yak | 1,924,882,809 | 15,043,644 | 0.78% |
| Taurine cattle VS Zebu cattle | 1,924,882,809 | 7,440,330 | 0.39% |
| American bison VS European bison | 1,924,882,809 | 7,110,502 | 0.37% |

^a^ The heterozygous sites in the genome sequence of Zebu cattle were replaced with random selected base of two possibilities.

**Table S13: Summary statistics of gene families in 7 species.**

| **Species** | **Total genes** | **Genes in families** | **Unclustered genes** | **Families** | **Unique families** | **Genes per family** | **Maximum gene family size** |
| --- | --- | --- | --- | --- | --- | --- | --- |
| **Taurine cattle** | 19,994 | 19,353 | 641 | 15,699 | 9 | 1.23 | 117 |
| **dog** | 19,856 | 18,201 | 1,655 | 14,878 | 40 | 1.22 | 108 |
| **horse** | 20,449 | 19,660 | 789 | 14,838 | 62 | 1.32 | 514 |
| **human** | 22,017 | 21,199 | 818 | 15,215 | 138 | 1.39 | 255 |
| **sheep** | 20,921 | 19,219 | 1,702 | 15,638 | 51 | 1.23 | 90 |
| **wisent** | 21,542 | 19,504 | 2,038 | 15,715 | 57 | 1.24 | 60 |
| **yak** | 22,282 | 20,150 | 2,132 | 16,174 | 58 | 1.25 | 121 |
| **All** | 147,061 | 137,286 | 9,775 | 17,610 | - | 7.80 | 1,265 |

**Table S14: Genes subject to positive selection in wisent.**

| GeneID | Descriptome |
| --- | --- |
| BB.scaffold1.00056 | zinc finger protein 526 prot_id:NP_001095467.1 Symbol:ZNF526 |
| BB.scaffold1.00062 | serine/arginine repetitive matrix protein 1-like prot_id:XP_003584089.2 |
| BB.scaffold10.00331 | synaptic Ras GTPase activating protein 1 prot_id:NP_001192395.1 Symbol:SYNGAP1 |
| BB.scaffold102.00509 | mucin 20, cell surface associated prot_id:XP_580797.6 Symbol:MUC20 |
| BB.scaffold1121.01226 | leukocyte immunoglobulin-like receptor, subfamily B (with TM and ITIM domains), member 3, transcript variant X1 prot_id:XP_005192909.1 Symbol:LILRB3 |
| BB.scaffold116.01444 | solute carrier family 4, sodium bicarbonate cotransporter, member 8, transcript variant X1 prot_id:XP_618089.7 Symbol:SLC4A8 |
| BB.scaffold1260.02094 | olfactory receptor, family 11, subfamily G, member 2 prot_id:XP_001252370.2 Symbol:OR11G2 |
| BB.scaffold137.02539 | methionine adenosyltransferase II, beta prot_id:NP_001039991.1 Symbol:MAT2B |
| BB.scaffold142.02859 | homeobox A3 prot_id:NP_001070293.1 Symbol:HOXA3 |
| BB.scaffold143.02890 | olfactory receptor, family 1, subfamily A, member 1-like prot_id:XP_600867.4 |
| BB.scaffold146.03044 | Ras association and DIL domains prot_id:NP_001157406.1 Symbol:RADIL |
| BB.scaffold150.03405 | solute carrier family 24 (sodium/potassium/calcium exchanger), member 1 prot_id:NP_777080.1 Symbol:SLC24A1 |
| BB.scaffold16.03960 | sialic acid binding Ig-like lectin 5-like prot_id:XP_588462.3 |
| BB.scaffold1766.04814 | olfactory receptor, family 51, subfamily L, member 1-like prot_id:XP_001249583.2 |
| BB.scaffold178.04844 | mitogen-activated protein kinase kinase kinase 13 prot_id:NP_001095323.1 Symbol:MAP3K13 |
| BB.scaffold198.05658 | translocase of outer mitochondrial membrane 20 homolog (yeast) prot_id:NP_001092653.1 Symbol:TOMM20 |
| BB.scaffold20.06028 | kelch-like 21 (Drosophila) prot_id:NP_001070279.1 Symbol:KLHL21 |
| BB.scaffold20.06212 | unc-93 homolog B1 (C. elegans) prot_id:NP_001180076.1 Symbol:UNC93B1 |
| BB.scaffold207.06450 | phosphodiesterase 6A, cGMP-specific, rod, alpha prot_id:NP_001001526.2 Symbol:PDE6A |
| BB.scaffold208.06504 | caspase 8 associated protein 2, transcript variant X3 prot_id:XP_005199457.1 Symbol:CASP8AP2 |
| BB.scaffold21.06566 | TNF receptor-associated factor 3 interacting protein 1 prot_id:NP_001178973.1 Symbol:TRAF3IP1 |
| BB.scaffold211.06669 | keratin-associated protein 10-4-like, transcript variant X2 prot_id:XP_005195799.1 |
| BB.scaffold211.06686 | bromodomain and WD repeat domain containing 1 prot_id:NP_001096644.1 Symbol:BRWD1 |
| BB.scaffold220.07034 | potassium voltage-gated channel, subfamily H (eag-related), member 6 prot_id:NP_001178323.1 Symbol:KCNH6 |
| BB.scaffold232.07360 | nucleus accumbens associated 1, BEN and BTB (POZ) domain containing prot_id:NP_001179541.1 Symbol:NACC1 |
| BB.scaffold24.07626 | myosin VB prot_id:XP_591875.5 Symbol:MYO5B |
| BB.scaffold240.07728 | potassium channel, subfamily K, member 1 prot_id:NP_001068675.1 Symbol:KCNK1 |
| BB.scaffold243.07820 | CDC42 binding protein kinase gamma (DMPK-like) prot_id:NP_001095972.1 Symbol:CDC42BPG |
| BB.scaffold253.08093 | diaphanous-related formin 3 prot_id:NP_001179543.1 Symbol:DIAPH3 |
| BB.scaffold266.08641 | frizzled family receptor 8 prot_id:XP_005194368.1 Symbol:FZD8 |
| BB.scaffold267.08698 | olfactory receptor, family 6, subfamily C, member 3-like prot_id:XP_001255218.2 |
| BB.scaffold28.09180 | GNAS complex locus, transcript variant 2 prot_id:NP_001258700.1 Symbol:GNAS |
| BB.scaffold282.09356 | CUGBP, Elav-like family member 4 prot_id:NP_001092538.1 Symbol:CELF4 |
| BB.scaffold292.09679 | parathyroid hormone 1 receptor prot_id:NP_001068800.1 Symbol:PTH1R |
| BB.scaffold300.10200 | uncharacterized LOC618217, transcript variant X3 prot_id:XP_005197067.1 |
| BB.scaffold325.10795 | BR serine/threonine kinase 2 prot_id:XP_615982.6 Symbol:BRSK2 |
| BB.scaffold331.11008 | olfactory receptor, family 12, subfamily D, member 2 prot_id:NP_001091852.1 Symbol:OR12D2 |
| BB.scaffold331.11015 | solute carrier family 12 (potassium/chloride transporters), member 7 prot_id:XP_003585295.2 Symbol:SLC12A7 |
| BB.scaffold344.11407 | SWI/SNF related, matrix associated, actin dependent regulator of chromatin, subfamily a, member 2 prot_id:NP_001092585.1 Symbol:SMARCA2 |
| BB.scaffold348.11488 | prolactin-related protein VI prot_id:NP_991347.1 Symbol:PRP6 |
| BB.scaffold35.11517 | PARP1 binding protein, transcript variant X1 prot_id:XP_005198381.1 Symbol:PARPBP |
| BB.scaffold356.11645 | uncharacterized LOC780976 prot_id:XP_001249346.4 |
| BB.scaffold356.11647 | olfactory receptor, family 10, subfamily C, member 1-like prot_id:XP_001255473.3 |
| BB.scaffold374.12073 | contactin associated protein 1 prot_id:NP_001193824.1 Symbol:CNTNAP1 |
| BB.scaffold387.12555 | asparaginase homolog (S. cerevisiae) prot_id:XP_581777.5 Symbol:ASPG |
| BB.scaffold39.12612 | uncharacterized LOC533455, transcript variant 2 prot_id:XP_869208.6 |
| BB.scaffold410.13261 | nitric oxide synthase 3 (endothelial cell) prot_id:NP_851380.2 Symbol:NOS3 |
| BB.scaffold413.13393 | olfactory receptor, family 2, subfamily A, member 2-like prot_id:XP_592549.4 |
| BB.scaffold47.14616 | neurofilament, heavy polypeptide, transcript variant X3 prot_id:XP_005195186.1 Symbol:NEFH |
| BB.scaffold473.14796 | ATP-binding cassette, sub-family B (MDR/TAP), member 4 prot_id:XP_003582118.1 Symbol:ABCB4 |
| BB.scaffold488.15048 | heat shock 70kDa protein 4-like, transcript variant 3 prot_id:XP_870867.6 Symbol:HSPA4L |
| BB.scaffold5.15349 | ATP-binding cassette, sub-family C (CFTR/MRP), member 4, transcript variant X2 prot_id:XP_593336.2 Symbol:ABCC4 |
| BB.scaffold509.15674 | CD97 molecule prot_id:NP_788834.1 Symbol:CD97 |
| BB.scaffold51.15684 | forkhead box P1 prot_id:NP_001077158.1 Symbol:FOXP1 |
| BB.scaffold531.15912 | ATPase, Na+/K+ transporting, alpha 3 polypeptide, transcript variant 1 prot_id:XP_606264.7 Symbol:ATP1A3 |
| BB.scaffold567.16468 | PTK2B protein tyrosine kinase 2 beta prot_id:NP_001095722.1 Symbol:PTK2B |
| BB.scaffold573.16552 | ATPase, Ca++ transporting, plasma membrane 2 prot_id:NP_001178174.1 Symbol:ATP2B2 |
| BB.scaffold6.17215 | mitogen-activated protein kinase 8 interacting protein 3 prot_id:NP_001069564.2 Symbol:MAPK8IP3 |
| BB.scaffold6.17382 | intestinal alkaline phosphatase VII, transcript variant X2 prot_id:XP_594531.4 |
| BB.scaffold655.17943 | euchromatic histone-lysine N-methyltransferase 1 prot_id:NP_001092511.1 Symbol:EHMT1 |
| BB.scaffold665.18085 | olfactory receptor, family 8, subfamily B, member 3-like prot_id:XP_005197353.1 |
| BB.scaffold68.18234 | RAN binding protein 17, transcript variant X3 prot_id:XP_005196167.1 Symbol:RANBP17 |
| BB.scaffold73.18908 | hyperpolarization activated cyclic nucleotide-gated potassium channel 3 prot_id:NP_001179207.1 Symbol:HCN3 |
| BB.scaffold744.19064 | ring finger protein 113A-like prot_id:XP_005197342.1 |
| BB.scaffold761.19208 | olfactory receptor, family 6, subfamily C, member 76-like, transcript variant X2 prot_id:XP_005193688.1 |
| BB.scaffold78.19390 | EGF-like module-containing mucin-like hormone receptor-like 4-like prot_id:XP_005198975.1 |
| BB.scaffold78.19446 | perilipin 4, transcript variant X10 prot_id:XP_588231.5 Symbol:PLIN4 |
| BB.scaffold78.19505 | lamin B2 prot_id:NP_001263282.1 Symbol:LMNB2 |
| BB.scaffold78.19523 | septin 8 prot_id:NP_001069698.1 Symbol:SEPT8 |
| BB.scaffold87.20379 | collagen, type I, alpha 1 prot_id:NP_001029211.1 Symbol:COL1A1 |
| BB.scaffold887.20470 | transcription factor binding to IGHM enhancer 3 prot_id:NP_001069279.2 Symbol:TFE3 |
| BB.scaffold888.20474 | maltase-glucoamylase, intestinal-like prot_id:XP_003582182.2 |
| BB.scaffold901.20663 | olfactory receptor, family 6, subfamily C, member 3-like prot_id:XP_001255375.1 |
| BB.scaffold921.20797 | olfactory receptor, family 7, subfamily A, member 17-like prot_id:XP_001251951.2 |
| BB.scaffold97.21347 | collagen alpha-1(XXVII) chain A-like prot_id:XP_005195701.1 |

**Table S15: Enriched gene ontology of positively selected genes.**

| **GO** | **Type** | **Function** | **Fdr** |
| --- | --- | --- | --- |
| GO:0043266 | biological process | regulation of potassium ion transport | 4.17E-09 |
| GO:0006813 | biological process | potassium ion transport | 1.36E-06 |
| GO:0048771 | biological process | tissue remodeling | 3.50E-06 |
| GO:0007172 | biological process | signal complex assembly | 4.45E-05 |
| GO:0030826 | biological process | regulation of cGMP biosynthetic process | 4.45E-05 |
| GO:0045453 | biological process | bone resorption | 1.43E-04 |
| GO:0015405 | molecular function | P-P-bond-hydrolysis-driven transmembrane transporter activity | 3.84E-04 |
| GO:0071805 | biological process | potassium ion transmembrane transport | 8.91E-04 |
| GO:0033267 | cellular component | axon part | 1.35E-03 |
| GO:0031175 | biological process | neuron projection development | 3.64E-02 |

**References**

1. Jirimutu, Wang Z, Ding G, Chen G, Sun Y, Sun Z, Zhang H, Wang L, Hasi S, Zhang Y *et al*: **Genome sequences of wild and domestic bactrian camels**. *Nat Commun* 2012, **3**:1202.

2. Groenen MA, Archibald AL, Uenishi H, Tuggle CK, Takeuchi Y, Rothschild MF, Rogel-Gaillard C, Park C, Milan D, Megens HJ *et al*: **Analyses of pig genomes provide insight into porcine demography and evolution**. *Nature* 2012, **491**(7424):393-398.

3. Fang X, Mou Y, Huang Z, Li Y, Han L, Zhang Y, Feng Y, Chen Y, Jiang X, Zhao W *et al*: **The sequence and analysis of a Chinese pig genome**. *Gigascience* 2012, **1**(1):16.

4. Scally A, Dutheil JY, Hillier LW, Jordan GE, Goodhead I, Herrero J, Hobolth A, Lappalainen T, Mailund T, Marques-Bonet T *et al*: **Insights into hominid evolution from the gorilla genome sequence**. *Nature* 2012, **483**(7388):169-175.

5. Zhou X, Sun F, Xu S, Fan G, Zhu K, Liu X, Chen Y, Shi C, Yang Y, Huang Z *et al*: **Baiji genomes reveal low genetic variability and new insights into secondary aquatic adaptations**. *Nat Commun* 2013, **4**:2708.

6. Yim HS, Cho YS, Guang X, Kang SG, Jeong JY, Cha SS, Oh HM, Lee JH, Yang EC, Kwon KK *et al*: **Minke whale genome and aquatic adaptation in cetaceans**. *Nat Genet* 2014, **46**(1):88-92.

7. Dong Y, Xie M, Jiang Y, Xiao N, Du X, Zhang W, Tosser-Klopp G, Wang J, Yang S, Liang J *et al*: **Sequencing and automated whole-genome optical mapping of the genome of a domestic goat (Capra hircus)**. *Nature biotechnology* 2013, **31**(2):135-141.

8. Ge RL, Cai Q, Shen YY, San A, Ma L, Zhang Y, Yi X, Chen Y, Yang L, Huang Y *et al*: **Draft genome sequence of the Tibetan antelope**. *Nat Commun* 2013, **4**:1858.

9. Li M, Tian S, Jin L, Zhou G, Li Y, Zhang Y, Wang T, Yeung CK, Chen L, Ma J *et al*: **Genomic analyses identify distinct patterns of selection in domesticated pigs and Tibetan wild boars**. *Nat Genet* 2013, **45**(12):1431-1438.

10. Lewis NE, Liu X, Li Y, Nagarajan H, Yerganian G, O'Brien E, Bordbar A, Roth AM, Rosenbloom J, Bian C *et al*: **Genomic landscapes of Chinese hamster ovary cell lines as revealed by the Cricetulus griseus draft genome**. *Nature biotechnology* 2013, **31**(8):759-765.

11. Seim I, Fang X, Xiong Z, Lobanov AV, Huang Z, Ma S, Feng Y, Turanov AA, Zhu Y, Lenz TL *et al*: **Genome analysis reveals insights into physiology and longevity of the Brandt's bat Myotis brandtii**. *Nat Commun* 2013, **4**:2212.

12. Fan Y, Huang ZY, Cao CC, Chen CS, Chen YX, Fan DD, He J, Hou HL, Hu L, Hu XT *et al*: **Genome of the Chinese tree shrew**. *Nat Commun* 2013, **4**:1426.

13. Jiang Y, Xie M, Chen W, Talbot R, Maddox JF, Faraut T, Wu C, Muzny DM, Li Y, Zhang W *et al*: **The sheep genome illuminates biology of the rumen and lipid metabolism**. *Science* 2014, **344**(6188):1168-1173.

14. Carneiro M, Rubin CJ, Di Palma F, Albert FW, Alfoldi J, Barrio AM, Pielberg G, Rafati N, Sayyab S, Turner-Maier J *et al*: **Rabbit genome analysis reveals a polygenic basis for phenotypic change during domestication**. *Science* 2014, **345**(6200):1074-1079.

15. Peng X, Alfoldi J, Gori K, Eisfeld AJ, Tyler SR, Tisoncik-Go J, Brawand D, Law GL, Skunca N, Hatta M *et al*: **The draft genome sequence of the ferret (Mustela putorius furo) facilitates study of human respiratory disease**. *Nature biotechnology* 2014, **32**(12):1250-1255.

16. Fang X, Nevo E, Han L, Levanon EY, Zhao J, Avivi A, Larkin D, Jiang X, Feranchuk S, Zhu Y *et al*: **Genome-wide adaptive complexes to underground stresses in blind mole rats Spalax**. *Nat Commun* 2014, **5**:3966.

17. Zhou X, Wang B, Pan Q, Zhang J, Kumar S, Sun X, Liu Z, Pan H, Lin Y, Liu G *et al*: **Whole-genome sequencing of the snub-nosed monkey provides insights into folivory and evolutionary history**. *Nat Genet* 2014, **46**(12):1303-1310.

18. **The common marmoset genome provides insight into primate biology and evolution**. *Nat Genet* 2014, **46**(8):850-857.

19. Carbone L, Harris RA, Gnerre S, Veeramah KR, Lorente-Galdos B, Huddleston J, Meyer TJ, Herrero J, Roos C, Aken B *et al*: **Gibbon genome and the fast karyotype evolution of small apes**. *Nature* 2014, **513**(7517):195-201.

20. Keane M, Semeiks J, Webb AE, Li YI, Quesada V, Craig T, Madsen LB, van Dam S, Brawand D, Marques PI *et al*: **Insights into the evolution of longevity from the bowhead whale genome**. *Cell reports* 2015, **10**(1):112-122.

21. Huang J, Zhao Y, Bai D, Shiraigol W, Li B, Yang L, Wu J, Bao W, Ren X, Jin B *et al*: **Donkey genome and insight into the imprinting of fast karyotype evolution**. *Sci Rep* 2015, **5**:14106.

22. Meyer WK, Venkat A, Kermany AR, van de Geijn B, Zhang S, Przeworski M: **Evolutionary history inferred from the de novo assembly of a nonmodel organism, the blue-eyed black lemur**. *Mol Ecol* 2015, **24**(17):4392-4405.

23. Fitak RR, Mohandesan E, Corander J, Burger PA: **The de novo genome assembly and annotation of a female domestic dromedary of North African origin**. *Molecular ecology resources* 2016, **16**(1):314-324.

24. Agaba M, Ishengoma E, Miller WC, McGrath BC, Hudson CN, Bedoya Reina OC, Ratan A, Burhans R, Chikhi R, Medvedev P *et al*: **Giraffe genome sequence reveals clues to its unique morphology and physiology**. *Nat Commun* 2016, **7**:11519.

25. Gordon D, Huddleston J, Chaisson MJ, Hill CM, Kronenberg ZN, Munson KM, Malig M, Raja A, Fiddes I, Hillier LW *et al*: **Long-read sequence assembly of the gorilla genome**. *Science* 2016, **352**(6281):aae0344.
