# Supplementary material for: The genome sequence of the wisent (Bison bonasus)
Source: Gigascience. 2017 Mar 10;6(4):1–5. doi: 10.1093/gigascience/gix016 (PMC5530314; doi:10.1093/gigascience/gix016)
Supplement: Response_to_reviewers_comments_revision_1.pdf [file gix016_Response_to_reviewers_comments_revision_1.pdf]

Dear Editor
Here we submit the revised manuscript entitled ‘The genome sequence of the wisent (Bison bonasus)’. We thank you and the reviewers for critical feedback on the previous version of our manuscript. We are very grateful to the referees for their rigorous comments, which have helped us improve our manuscript. Specifically, we have submitted the assembly pipeline and log file to GitHub (Including BUSCO analysis). We also re-analyzed and investigated the deeper synteny relationship between wisent and cattle. Below you will find our point-by-point responses to the reviewers’ comments and questions.
We hope that this revision is suitable for publication. If you have more questions, please do not hesitate to contact us.

Best wishes
Sincerely yours,
Jianquan

Reply to reviewers
To review #1,
I am still quite skeptical about the quality of this assembly, particularly given the authors' exceptionally high BUSCO scores! They suggest that their wisent genome assembly surpasses the BUSCO scores for the human genome (90% complete) and the improved cattle genome (btau8; 84% complete), and they do not provide (a) the BUSCO version number and (b) the command line arguments for BUSCO analysis. For ranges of BUSCO scores for domestic livestock reference assemblies, please see: Seeman et al.. 2015. Bioinformatics and Biological Insights. An excellent de novo short read assembly (without manual curation) is likely to receive a BUSCO score of 70 - 80%.
In order to address this concern, I would like to see the authors provide the following information:
* The command line arguments and version of BUSCO used.
* A discussion of why (if the scores are correct) they have higher BUSCO scores than the curated Human reference assembly
Reply:
We agree with the reviewer’s comments, which reflect our misunderstanding of the most appropriate BUSCO score:
First, there are three running modes of BUSCO (v1.22): ‘genome’ (genome assembly), ‘protein’ (gene set/proteome) and ‘transcriptome’. In the ‘all’ mode BUSCO itself predicts gene structure while in the ‘protein’ mode BUSCO adopts a given gene set, in our case based on our annotation. One obvious advantage of the ‘all’ mode is that the quality of the authors’ annotation is variable and the score based on BUSCO gene prediction will be better comparable across different studies. However, we found that not all genes were predicted correctly by BUSCO in comparison with our annotated gene (Table S4). Thus, we only gave the result of the ‘protein’ mode in the manuscript. In order to obtain a more meaningful comparison with other studies, the results of both ‘genome’ mode and ‘protein’ modes are listed in the revised manuscript.
Secondly, instead of the mentioning most-used BUSCO score based on complete matching with the orthologs, we also considered incomplete gene assemblies. So, we have replaced “99% of 3023 single-copy orthologs of the vertebrate lineages were assembled” (‘protein’ mode)” by “the BUSCO score (‘genome’ mode) is 83.4% in wisent”.
* Inclusion of the UMD3.1 cattle reference assembly, and the OAR3 sheep reference assembly on the FRC curve plots
* provide version numbers and command line arguments for all software programs used, so that the assembly process can be reproducible

Reply:
We have added the FRC curve of these two assemblies and added version numbers and command line arguments for all programs used. In addition, we have also released our assembly pipeline, assessment pipeline and software report to GitHub ‘Availability of supporting data’ for further studies.
To reviewer #2,
The authors performed synteny analysis in comparison with related species. However, they reported the result only from chromosome 1 of cattle as the Supp. Figure 7. It would be better if the authors also provide a summary of synteny analysis results from all chromosomes.
Reply:
According to the suggestion, we plotted and summarized the synteny relationships within the whole genome of cattle and wisent (Figure S8 and Table S11). We used circos and custom scripts (submitted to GitHub) instead of mcscan for plotting Figure S8.

In the synteny analysis provided in the Supp. Figure 7, the portions of scaffold211 and scaffold154 are mapped to different and dispersed positions in cattle chromosome1. Does this represent real transpositions between the two species, or misassembly in one of the species assembly? This needs to be discussed. I believe the authors can find more similar cases from other cattle chromosomes.
Reply:
The corresponding relationship between cattle and wisent in the Supp. Figure 7 in our previous manuscript was analyzed by mcscan, which makes use of the information of ortholog genes, but appeared to generate errors in gene prediction and ortholog identification. In the present version, we investigated the synteny alignment directly with circos.
The synteny alignment had been re-analyzed. We now have used the more accurate cattle genome assembly UMD 3.1 instead of Btau 4.6.1 except for the Y chromosome. We used ‘last’ instead of ‘lastz’, in order to handle repetitive elements and to obtain more comprehensive alignments. The breakpoints caused by transposition events or by assembly or synteny errors in the alignment were classified and compared between different regions of the genome. We also found that the density of the breakpoints is lower in longer scaffolds. All of these results were discussed in the ‘Comparative analysis’ section of the revised manuscript.

In this revision, the number of genes was changed from 22,254 to 21,542. What happened?
Reply:
The protein sequences of cattle used in the previous version of gene prediction were from Btau4.6.1, while we have used UMD3.1 for the revised version.

To reviewer 3#,
Figure S6: I believe the one of the labels in the figure should be "Intron" and not "Introl length"
Reply: corrected.
Table S1: The column label is "sequence coverate" and should be "sequence coverage"
Reply: corrected.
Page 1
Abstract ln 21: "genomes reproduces the close relationship of bison and wisent" not if bison and wisent
Reply: corrected.
Abstract1 ln 30-34: This sentence needs to be restated to either "These results will be" or "The results will be" and the "evolutionary history of the bovine species" is still not correct.
Reply: corrected.
Data Description Background ln 46: "Wisent is an impressively mammal" perhaps "impressive" or another term
Reply: corrected.
Page 2
Sequencing ln 2: How old and when was the wisent sample obtained? The authors state in the abstract that the wisent was rescued 80 years ago from 12 founding individuals so some confusion could occur that they sampled a wisent from 80 years ago and was one of the 12 founding animals. I do not think that was the case but more background on the sample would be nice.

Reply: Thanks for your advice, we have added the sample date.
Sequencing ln 15: A word seems to be missing that connects the "genomic DNA was … and circularized"
Reply: corrected.
Assembly ln 34: Is it supposed to be >2kb or >2kp?
Reply: corrected.
Page 4:
Ln 6: Compared the gene set of" needs to be changed to "Comparison of the gene sets of"… showed there were no…
Reply: corrected.
Ln 15 "we found no pathway show significant difference" should be no pathways
Reply: corrected.
Ln 18-19: "contain significant more genes in yak" should change to "contain significantly more…"
Reply: corrected.
Ln 21: "and the gene composition were conservative in these three species" change conservative to conserved.
Reply: corrected.
Comparative Analysis ln 26: "We downloaded the nuclear genomes of including yak…." Remove the "of including" and change to for
Reply: This sentence had been re-written to “The synteny alignments within these six species” in the first sentence of the second paragraph of “Comparative analysis”.
Comparative Analysis ln 31: add space between spanning and 1,055 Mb.
Reply: The information of synteny alignments had been removed into table S11.
Comparative Analysis ln 40: still has bovine species
Reply: corrected.

Clarification on the animal chosen to represent the wisent genome is important not only to better understand when the sample was collected but what that could mean for what is found in the genome. As with North American bison, wisent can breed with domestic cattle and create hybrids that are hard to distinguish if back crossed by just looking at them. What precautions were taken to ensure that the animal chosen was not a hybrid, with either cattle, or North American bison?
Reply:
The phenotype of our sample was carefully checked before sampling. The wisent population of the Kennemerduinen originates from the pure Lowland lineage and never had any opportunity of crossbreeding. Our sample was a bull calf born in the Kraansvlak herd (a part of the Kennemerduinen and the official name of the herd). The mother has been captured in Bialowieza and the founder bull was Podryw from Przyna, both from the Lowland line. Since recording started, the herd never has been in contact with either American bison or domestic cattle. The mitochondrial sequence of this wisent was further found to be almost identical to published wisent mtDNA sequences (Fig S2). If recent hybridization occurred between wisent and either cattle or American bison, isolated genomic regions would exhibit extremely small genetic distance to cattle or bison, respectively. We therefore applied sliding window method (50 Kb) to detect in our wisent nuclear genome regions with significantly abnormal genetic distance between wisent and cattle or American bison, but found no signal of recent hybridization (Fig S11).
To reviewer 4#,
The revision has addressed my concerns. Hopefully the editorial office can help edit their abstract. It seems that one sentence needs to be polished.
Reply:
We appreciate the reviewer’s positive comments. We have re-examined our manuscript and improved the clarity.
