# Supplementary material for: The genome sequence of the wisent (Bison bonasus)
Source: Gigascience. 2017 Mar 10;6(4):1–5. doi: 10.1093/gigascience/gix016 (PMC5530314; doi:10.1093/gigascience/gix016)
Supplement: Response_to_reviewer_comments_Original_Submission.pdf [file gix016_Response_to_reviewer_comments_Original_Submission.pdf]

GIGA-D-16-00043
Draft genome of European bison (wisent), Bison bonasus
Kun Wang; Lizhong Wang; Johannes A. Lenstra; Jianbo Jian; Quanjun Hu; Yongzhi Yang; Deyong Lai; Qiang Qiu; Tao Ma; Richard Abbott; Jianquan Liu, Ph.D
GigaScience

Dear Prof. Liu,

Your manuscript "Draft genome of European bison (wisent), Bison bonasus" (GIGA-D-16-00043) has been assessed by our reviewers. Although it is of interest, we are need a bit more work to consider it for publication in its current form. The reviewers have raised a number of points which we believe would improve the manuscript and may allow a revised version to be published in GigaScience. In particular there need to be some more metrics/comparisons, and improvements to the language. We copy edit our papers before publication, but there were some embarrassing problems like calling it "Daft genome".

Their reports, together with any other comments, are below. Please also take a moment to check our website at http://giga.edmgr.com/ for any additional comments that were saved as attachments.

If you are able to fully address these points, we would encourage you to submit a revised manuscript to GigaScience. Once you have made the necessary corrections, please submit online at:

http://giga.edmgr.com/

If you have forgotten your username or password please use the "Send Login Details" link to get your login information. For security reasons, your password will be reset.

Please include a point-by-point within the 'Response to Reviewers' box in the submission system. Please ensure you describe additional experiments that were carried out and include a detailed rebuttal of any criticisms or requested revisions that you disagreed with. Please also ensure that your revised manuscript conforms to the journal style, which can be found in the Instructions for Authors on the journal homepage.

The due date for submitting the revised version of your article is 17 Oct 2016.

I look forward to receiving your revised manuscript soon.

Best wishes,

Scott Edmunds, Ph.D.
GigaScience
www.gigasciencejournal.com

Reviewer reports:

Reviewer #1: Summary: In this data release, Wang et al. detail how they assembled sequence data from the European Bison, also known as the Wisent. They have uploaded data and summary statistics from their assembly for public use and it appears that their assembly was constructed entirely by mixed insert length Illumina WGS libraries generated from Wisent DNA. While their contig N50 is within the typical range of short-read assemblies, their scaffold N50 is abnormally high for an assembly that does not use fosmid end- or BAC end-sequence data. I would recommend one or two more summary tests of this data prior to submission just to assist the reader in assessing the quality of this assembly.
Reply: We appreciate the reviewer’s comments on this manuscript and we found all the suggestions to be highly useful for improving our manuscript.

Recommended revisions:

* Abstract: European Bison are members of the Bovidae family, not the bovine species. The authors make this mistake frequently in the text and this is a very important distinction as they do not have direct relations with Bos taurus subspecies.
Reply: We have changed the word ‘bovine species’ into ‘Bovini tribe’, which would be more precision.

* Data description pg 1: ln 51: Again, Wisent are members of the Bovidae family.
Reply: Done.

* Summary table S1: It would be useful to list which libraries were paired-end and which libraries were mate-paired.
Reply: The detailed information of libraries is now included in table S1.

* Page 2 ln 39: How many gaps were closed by "Gapcloser?"
Reply: We have added the related description to the revised manuscript (in the second paragraph of the “Assembly” section).

* Page 2 ln 50: This is an incredible Scaffold N50 size for a genome that only uses mate-paring for scaffolding! Compared to a similar short-read assembly that used a similar strategy and tools (Goat, Dong et al. 2013), the scaffold N50 for Wisent is apparently 2 times the N50 of the initial Goat scaffolds (pre-fosmid end scaffolding). The authors do not need to describe this in incredible detail, but some reason why their assembly is of such high N50 might be needed.
Reply: The scaffold N50 size of an assembly from NGS sequencing is largely effected by the strategy of library constructing, sequencing quality, assembly software and parameters. The assembly algorithm and software have been largely improved in recent year. On the one hand, the complex genome with high heterozygosity could be resolved with new algorithms [1]. On the other hand, the genome assembly quality could also be great improved even with original data [2]. Overall, the improved sequencing technology, assembly algorithm and optimized parameter tuning resulted a better performance in latest published genomes (table S3), many of which were larger than that in wisent. In addition, we have performed several quality test including BUSCO, CEGMA, which proved that the completeness and continuity of wisent genome were comparable to relative species.
1. Kajitani R, Toshimoto K, Noguchi H, Toyoda A, Ogura Y, Okuno M, Yabana M, Harada M, Nagayasu E, Maruyama H et al: Efficient de novo assembly of highly heterozygous genomes from whole-genome shotgun short reads. Genome research 2014, 24(8):1384-1395.
2. Luo R, Liu B, Xie Y, Li Z, Huang W, Yuan J, He G, Chen Y, Pan Q, Liu Y et al: SOAPdenovo2: an empirically improved memory-efficient short-read de novo assembler. Gigascience 2012, 1(1):18.

* Summary table S2: Can you please include total contig and scaffold counts in this table?
Reply: We have added contig and scaffold counts information in table S2.

* Page 2 ln 51: It can be very difficult to assess assembly quality; however, I think that there are some rather quick analyses that can be performed on this dataset that will give the reader some sense of the quality of the resulting scaffolds. I recommend calculating a BUSCO score for your scaffolds and generating an FRC curve for the assembly.
Reply: We have performed BUSCO analysis and generated FRC curve for this assembly and the related information is listed in table S3 and fig. S2.

* Page 3 ln 5: Replace this sentence: "We found 47.3% in the genome of wisent is composed of repetitive elements" to the following: "We found that 47.3% of our wisent assembly is composed of repetitive elements"
Reply: Fixed.

* Page 3 ln 34: Again, replace "bovine" with the "Bovidae family"
Reply: Fixed.

* Availability of supporting data: It would be incredibly helpful if you provided the Bioproject ID, and the three BioSample IDs associated with this project. NCBI's databases supply you with several different accession IDs and it makes it easier for the reader if all accessions are provided up front.
Reply: We have added related information in the manuscript (in the “Availability of supporting data” section), we also added the accession id for each library in table S1.

Reviewer #2: This article describes the draft genome of European bison, which is one of very important endangered animals. The authors collected samples from one of rescued individual approximately 80 years ago, sequenced them using the next-generation sequencers, and assembled them using the state-of-the-art genome assembly technologies. The data reported in this article will be used as a very valuable resource for many studies for the bovine species, artiodactyla, and even for entire mammals.
However, the quality of this article would be much improved if the following issues are addressed.
Reply: We are grateful for these positive comments and have addressed all of the points raised by the reviewer.

1. Once a de-novo genome sequence of a new species is created, it is important to compare them with related species. So it would be better if the authors compare the composition of repeats and protein coding genes with related species, like yak and cattle. In addition, synteny analysis with the genomes of related species would be also interesting.
Reply: According to the review’s advice, we have done the following analysis and added them to the manuscript: (a) the composition of different class of repeat sequences (DNA, LINE, SINE, LTR) were compared between wisent and yak; (b) the distribution of gene length, exon length, etc. were compared between wisent, yak and cattle; (c) the KEGG pathway information of wisent, yak and cattle were annotated and compared with the latest database; (d) the 1:1 orthologous sequences were obtained and analyzed within six species (cattle, zebu cattle, yak, buffalo, bison, wisent); (e) the gene cluster analysis were performed within 8 species and the species-specific genes were identified; (f) the positive selected genes were identified by branch-site model with PAML; (g) the genetic distance and phylogeny relationship within six species were analyzed with the genomic synteny alignment.

2. In addition to the length of a genome assembly, the quality in terms of missassembly is also important. It would be better if the authors add results from additional checking for the quality of their genome assembly using some tools and resources, like the CEGMA pipeline.
Reply: We have performed CEGMA analysis and BUSCO analysis among three species of Bovidae family.

3. In Table S1, it would be much better for readers if additional information, like read length, sequence and physical coverage, and similar numbers before and after QC, is provided.
Reply: We have added these information in table S1. However, we had only preserved the reads after quality filtering because there is not enough storage space.

4. The data is already available in their website, and it is very nice. But I tried to search for sequencing reads in the NCBI website using their project ID, and failed. Please check the availability of the data in the NCBI website again.
Reply: The reads will be public after the manuscript is accepted.

Reviewer #3: On page 1 in the data description line 59 the authors state the "Wisent is now listed as threated", perhaps threatened is the word to use.
Reply: We have fixed this in the manuscript.

Why was the tongue used for genetic material?
Reply: This is because we can only get these materials.

This is an informative paper on the wisent assembly. However, more comparison to the yak and cattle

genome could be informative on the assembly that was done. For instance comparing, N50 sizes and then also the predicted number of genes for the wisent. This could verify that the assembly is complete and thorough by using this comparison.
Reply: We have added more comparisons between wisent with yak and cattle in the manuscript (third paragraph of section “Assembly”, page 3, line 29; third paragraph of section “Annotation”, page 5, line 5) and table 1.

Reviewer #4: Comments on Daft genome of European bison (wisent), Bison bonasus by Wang et al.

Wang and colleagues report the first, but the draft genome of Europen bison. The material was collected from a dead male animal for DNA extraction. Both long and short size libraries were prepared and sequenced using the Illumina platform. As results, the authors were able to assemble 2.58 Gb of sequences for the species with over 120x of sequencing depth. Overall, this paper presents a simple data report.

Like cow and yak, the America bison was found to have 60 chromosomes (http://www.wow.com/wiki/List_of_number_of_chromosomes_of_various_organisms). How about the European bison then? As such, can authors map these scaffolds to chromosomes?
Reply: The chromosome number of European bison is also 60, which had been studied by Melander in 1959 [1]. However, we don’t have a physical genetic map, so we cannot assemble the scaffolds to chromosomes. Instead, we performed a synteny analysis which mapped these scaffolds to the chromosomes of cattle (Fig. S7).

1. Melander Y: The mitotic chromosomes of some cavicorn mammals (Bos Taurus L., Bison Bonasus L. and Ovis Aries L.). Hereditas 1959, 45(4):649-664.

Several draft genomes of ruminant species are available. Can this work fill some gaps in determination of evolutionary relationships among bovini?
Reply: Phylogenetic origin of wisent based on present molecular evidence remain disputed. Wisent and bison (Bison bison) from North America are morphologically similar and completely cross-fertile with similar Y-chromosomal as well as autosomal DNA sequences. However, the mitochondrial DNA (mtDNA) genome of wisent consistently clusters in phylogenies with the mtDNA genome of ancestors of the taurine and zebu cattle lineage. In this study, we reconstructed the phylogeny relationship with the new wisent genome, which also support that European bison and American bison are sister species. The speciation pattern and evolutionary diversification of the bovine species need further detailed examination based on more population genomic data through re-sequencing genomes of more individuals.

Did the authors find any species-specific genes in wisent?
Reply: We have identified 57 unique gene families and 2,038 unclustered genes in wisent using OrthoMCL and related information was added in the manuscript (second paragraph in ‘Comparative analysis’ section, page 5, line 22) and supplementary files (Table S10, Fig. S10).

Any transcriptome data to support those non-coding genes discovered in the study? How many non-coding sequences were derived from the coding genes?
Reply: The non-coding genes we identified here were small RNAs (miRNA, tRNA and rRNA) other than long non-coding RNA originated from coding genes. Due to the fact that mRNA with Poly(A) was enriched in the process of constructing cDNA libraries, there was no transcript evidence for these small RNAs.

Are there any reads related to the mitochondrial genome in the species?
Reply: We have extracted the reads related to the mitochondrial genome and performed an assembly (http://me.lzu.edu.cn/yak/).

Can the authors compile a list of potential SNPs for the species?
Reply: The SNPs and InDels (Insertions and Deletions) were added to the yak genome database (http://me.lzu.edu.cn/yak/) for downloading.

What does this work contribute to genome sciences and communities?
Reply: (1) European bison is a typical mammal that experienced several bottleneck. The genome sequences of this species is the basis for studying the bottleneck and its impact on genomic diversity and accumulation of harmful mutations. Our study would also be useful for researchers for further protection of this species.
(2) The phylogeny relationship and the speciation process of Bovini tribe has not been well reconstructed and our study would provide essential data for further analysis.

--
Please also take a moment to check our website at for any additional comments that were saved as attachments. Please note that as GigaScience has a policy of open peer review, you will be able to see the names of the reviewers.
