# Supplementary material for: The genome sequence of the wisent (Bison bonasus)
Source: Gigascience. 2017 Mar 10;6(4):1–5. doi: 10.1093/gigascience/gix016 (PMC5530314; doi:10.1093/gigascience/gix016)
Supplement: Response_to_reviewer_comments_Revision_2.pdf [file gix016_Response_to_reviewer_comments_Revision_2.pdf]

To reviewer 2#,
The authors have addressed all my concerns in this revision. One minor issue is the missing of legend in Supplementary Figure S8. Please add the species names in the figure (left: wisent, right: cattle).
Reply:
We appreciate the reviewer’s positive comments on our work. We have added legend of species name in Figure S8.
