# Supplementary material for: The genome sequence of the wisent (Bison bonasus)
Source: Gigascience. 2017 Mar 10;6(4):1–5. doi: 10.1093/gigascience/gix016 (PMC5530314; doi:10.1093/gigascience/gix016)
Supplement: Reviewer_1_Report_(Original_Submission).pdf [file gix016_Reviewer_1_Report_(Original_Submission).pdf]

Reviewer Report

Title: "The genome sequence of the wisent (Bison bonasus)"

Version: Original Submission **Date:** 7/8/2016

Reviewer name: Derek Bickhart

Reviewer Comments to Author:

Summary: In this data release, Wang et al. detail how they assembled sequence data from the European Bison, also known as the Wisent. They have uploaded data and summary statistics from their assembly for public use and it appears that their assembly was constructed entirely by mixed insert length Illumina WGS libraries generated from Wisent DNA. While their contig N50 is within the typical range of short-read assemblies, their scaffold N50 is abnormally high for an assembly that does not use fosmid end- or BAC end-sequence data. I would recommend one or two more summary tests of this data prior to submission just to assist the reader in assessing the quality of this assembly.

Recommended revisions:

* Abstract: European Bison are members of the Bovidae family, not the bovine species. The authors make this mistake frequently in the text and this is a very important distinction as they do not have direct relations with Bos taurus subspecies.

* Data description pg 1: ln 51: Again, Wisent are members of the Bovidae family.

* Summary table S1: It would be useful to list which libraries were paired-end and which libraries were mate-paired.

* Page 2 ln 39: How many gaps were closed by "Gapcloser?"

* Page 2 ln 50: This is an incredible Scaffold N50 size for a genome that only uses mate-paring for scaffolding! Compared to a similar short-read assembly that used a similar strategy and tools (Goat, Dong et al. 2013), the scaffold N50 for Wisent is apparently 2 times the N50 of the initial Goat scaffolds (pre-fosmid end scaffolding). The authors do not need to describe this in incredible detail, but some reason why their assembly is of such high N50 might be needed.

* Summary table S2: Can you please include total contig and scaffold counts in this table?

* Page 2 ln 51: It can be very difficult to assess assembly quality; however, I think that there are some rather quick analyses that can be performed on this dataset that will give the reader some sense of the quality of the resulting scaffolds. I recommend calculating a BUSCO score for your scaffolds and generating an FRC curve for the assembly.

* Page 3 ln 5: Replace this sentence: "We found 47.3% in the genome of wisent is composed of repetitive elements" to the following: "We found that 47.3% of our wisent assembly is composed of repetitive elements"

* Page 3 ln 34: Again, replace "bovine" with the "Bovidae family"

* Availability of supporting data: It would be incredibly helpful if you provided the Bioproject ID, and the three BioSample IDs associated with this project. NCBI's databases supply you with several different accession IDs and it makes it easier for the reader if all accessions are provided up front.

Level of Interest

Please indicate how interesting you found the manuscript: An article whose findings are important to those with closely related research interests

Quality of Written English

Please indicate the quality of language in the manuscript: Needs some language corrections before being published

Declaration of Competing Interests

Please complete a declaration of competing interests, considering the following questions:

- Have you in the past five years received reimbursements, fees, funding, or salary from an organisation that may in any way gain or lose financially from the publication of this manuscript, either now or in the future?
- Do you hold any stocks or shares in an organisation that may in any way gain or lose financially from the publication of this manuscript, either now or in the future?

- Do you hold or are you currently applying for any patents relating to the content of the manuscript?
- Have you received reimbursements, fees, funding, or salary from an organization that holds or has applied for patents relating to the content of the manuscript?
- Do you have any other financial competing interests?
- Do you have any non-financial competing interests in relation to this paper?

If you can answer no to all of the above, write 'I declare that I have no competing interests' below. If your reply is yes to any, please give details below.

I declare that I have no competing interests

I agree to the open peer review policy of the journal. I understand that my name will be included on my report to the authors and, if the manuscript is accepted for publication, my named report including any attachments I upload will be posted on the website along with the authors' responses. I agree for my report to be made available under an Open Access Creative Commons CC-BY license (http://creativecommons.org/licenses/by/4.0/). I understand that any comments which I do not wish to be included in my named report can be included as confidential comments to the editors, which will not be published.

I agree to the open peer review policy of the journal
