# Supplementary material for: The genome sequence of the wisent (Bison bonasus)
Source: Gigascience. 2017 Mar 10;6(4):1–5. doi: 10.1093/gigascience/gix016 (PMC5530314; doi:10.1093/gigascience/gix016)
Supplement: Reviewer_1_Report_(Revision_1).pdf [file gix016_Reviewer_1_Report_(Revision_1).pdf]

**Reviewer Report**

**Title:** "The genome sequence of the wisent (Bison bonasus)"

**Version:** Revision 1 **Date:** 11/4/2016

**Reviewer name:** Derek Bickhart

**Reviewer Comments to Author:**

I am still quite skeptical about the quality of this assembly, particularly given the authors' exceptionally high BUSCO scores! They suggest that their wisent genome assembly surpasses the BUSCO scores for the human genome (90% complete) and the improved cattle genome (btau8; 84% complete), and they do not provide (a) the BUSCO version number and (b) the command line arguments for BUSCO analysis. For ranges of BUSCO scores for domestic livestock reference assemblies, please see: Seeman et al.. 2015. Bioinformatics and Biological Insights. An excellent de novo short read assembly (without manual curation) is likely to receive a BUSCO score of 70 - 80%.

In order to address this concern, I would like to see the authors provide the following information:

* The command line arguments and version of BUSCO used.

* A discussion of why (if the scores are correct) they have higher BUSCO scores than the curated Human reference assembly

* Inclusion of the UMD3.1 cattle reference assembly, and the OAR3 sheep reference assembly on the FRC curve plots

* provide version numbers and command line arguments for all software programs used, so that the assembly process can be reproducible

**Level of Interest**

Please indicate how interesting you found the manuscript: An article whose findings are important to those with closely related research interests

**Quality of Written English**

Please indicate the quality of language in the manuscript: Acceptable

**Declaration of Competing Interests**

Please complete a declaration of competing interests, considering the following questions:

- Have you in the past five years received reimbursements, fees, funding, or salary from an organisation that may in any way gain or lose financially from the publication of this manuscript, either now or in the future?
- Do you hold any stocks or shares in an organisation that may in any way gain or lose financially from the publication of this manuscript, either now or in the future?
- Do you hold or are you currently applying for any patents relating to the content of the manuscript?
- Have you received reimbursements, fees, funding, or salary from an organization that holds or has applied for patents relating to the content of the manuscript?
- Do you have any other financial competing interests?
- Do you have any non-financial competing interests in relation to this paper?

If you can answer no to all of the above, write 'I declare that I have no competing interests' below. If your reply is yes to any, please give details below.

I declare that I have no competing interests

I agree to the open peer review policy of the journal. I understand that my name will be included on my report to the authors and, if the manuscript is accepted for publication, my named report including any attachments I upload will be posted on the website along with the authors' responses. I agree for my report to be made available under an Open Access Creative Commons CC-BY license (http://creativecommons.org/licenses/by/4.0/). I understand that any comments which I do not wish to be included in my named report can be included as confidential comments to the editors, which will not be published.

I agree to the open peer review policy of the journal
