# Supplementary material for: The genome sequence of the wisent (Bison bonasus)
Source: Gigascience. 2017 Mar 10;6(4):1–5. doi: 10.1093/gigascience/gix016 (PMC5530314; doi:10.1093/gigascience/gix016)
Supplement: Reviewer_2_Report_(Original_Submission).pdf [file gix016_Reviewer_2_Report_(Original_Submission).pdf]

**Reviewer Report**

**Title:** "The genome sequence of the wisent (Bison bonasus)"

**Version:** Original Submission **Date:** 7/9/2016

**Reviewer name:** Jaebum Kim

**Reviewer Comments to Author:**

This article describes the draft genome of European bison, which is one of very important endangered animals. The authors collected samples from one of rescued individual approximately 80 years ago, sequenced them using the next-generation sequencers, and assembled them using the state-of-the-art genome assembly technologies. The data reported in this article will be used as a very valuable resource for many studies for the bovine species, artiodactyla, and even for entire mammals.

However, the quality of this article would be much improved if the following issues are addressed.

1. Once a de-novo genome sequence of a new species is created, it is important to compare them with related species. So it would be better if the authors compare the composition of repeats and protein coding genes with related species, like yak and cattle. In addition, synteny analysis with the genomes of related species would be also interesting.

2. In addition to the length of a genome assembly, the quality in terms of missassembly is also important. It would be better if the authors add results from additional checking for the quality of their genome assembly using some tools and resources, like the CEGMA pipeline.

3. In Table S1, it would be much better for readers if additional information, like read length, sequence and physical coverage, and similar numbers before and after QC, is provided.

4. The data is already available in their website, and it is very nice. But I tried to search for sequencing reads in the NCBI website using their project ID, and failed. Please check the availability of the data in the NCBI website again.

**Level of Interest**

Please indicate how interesting you found the manuscript: An article of importance in its field

**Quality of Written English**

Please indicate the quality of language in the manuscript: Acceptable

**Declaration of Competing Interests**

Please complete a declaration of competing interests, considering the following questions:

- Have you in the past five years received reimbursements, fees, funding, or salary from an organisation that may in any way gain or lose financially from the publication of this manuscript, either now or in the future?
- Do you hold any stocks or shares in an organisation that may in any way gain or lose financially from the publication of this manuscript, either now or in the future?
- Do you hold or are you currently applying for any patents relating to the content of the manuscript?
- Have you received reimbursements, fees, funding, or salary from an organization that holds or has applied for patents relating to the content of the manuscript?
- Do you have any other financial competing interests?
- Do you have any non-financial competing interests in relation to this paper?

If you can answer no to all of the above, write 'I declare that I have no competing interests' below. If your reply is yes to any, please give details below.

I declare that I have no competing interests.

I agree to the open peer review policy of the journal. I understand that my name will be included on my report to the authors and, if the manuscript is accepted for publication, my named report including any attachments I upload will be posted on the website along with the authors' responses. I agree for my report to be made available under an Open Access Creative Commons CC-BY license (http://creativecommons.org/licenses/by/4.0/). I understand that any comments which I do not wish to be included in my named report can be included as confidential comments to the editors, which will not be published.

I agree to the open peer review policy of the journal
