# Supplementary material for: The genome sequence of the wisent (Bison bonasus)
Source: Gigascience. 2017 Mar 10;6(4):1–5. doi: 10.1093/gigascience/gix016 (PMC5530314; doi:10.1093/gigascience/gix016)
Supplement: Reviewer_2_Report_(Revision_1).pdf [file gix016_Reviewer_2_Report_(Revision_1).pdf]

Reviewer Report

Title: "The genome sequence of the wisent (Bison bonasus)"

Version: Revision 1 **Date:** 10/25/2016

Reviewer name: Jaebum Kim

Reviewer Comments to Author:

In this revision, the authors successfully addressed most of my comments except for the followings.

1. The authors performed synteny analysis in comparison with related species. However, they reported the result only from chromosome 1 of cattle as the Supp. Figure 7. It would be better if the authors also provide a summary of synteny analysis results from all chromosomes.

2. In the synteny analysis provided in the Supp. Figure 7, the portions of scaffold211 and scaffold154 are mapped to different and dispersed positions in cattle chromosome1. Does this represent real transpositions between the two species, or misassembly in one of the species assembly? This needs to be discussed. I believe the authors can find more similar cases from other cattle chromosomes.

3. In this revision, the number of genes was changed from 22,254 to 21,542. What happened?

Level of Interest

Please indicate how interesting you found the manuscript: An article of importance in its field

Quality of Written English

Please indicate the quality of language in the manuscript: Acceptable

Declaration of Competing Interests

Please complete a declaration of competing interests, considering the following questions:

- Have you in the past five years received reimbursements, fees, funding, or salary from an organisation that may in any way gain or lose financially from the publication of this manuscript, either now or in the future?
- Do you hold any stocks or shares in an organisation that may in any way gain or lose financially from the publication of this manuscript, either now or in the future?

- Do you hold or are you currently applying for any patents relating to the content of the manuscript?
- Have you received reimbursements, fees, funding, or salary from an organization that holds or has applied for patents relating to the content of the manuscript?
- Do you have any other financial competing interests?
- Do you have any non-financial competing interests in relation to this paper?

If you can answer no to all of the above, write 'I declare that I have no competing interests' below. If your reply is yes to any, please give details below.

I declare that I have no competing interests

I agree to the open peer review policy of the journal. I understand that my name will be included on my report to the authors and, if the manuscript is accepted for publication, my named report including any attachments I upload will be posted on the website along with the authors' responses. I agree for my report to be made available under an Open Access Creative Commons CC-BY license (http://creativecommons.org/licenses/by/4.0/). I understand that any comments which I do not wish to be included in my named report can be included as confidential comments to the editors, which will not be published.

I agree to the open peer review policy of the journal
