# Supplementary material for: The genome sequence of the wisent (Bison bonasus)
Source: Gigascience. 2017 Mar 10;6(4):1–5. doi: 10.1093/gigascience/gix016 (PMC5530314; doi:10.1093/gigascience/gix016)
Supplement: Reviewer_3_Report_(Original_Submission).pdf [file gix016_Reviewer_3_Report_(Original_Submission).pdf]

Reviewer Report

Title: "The genome sequence of the wisent (Bison bonasus)"

Version: Original Submission **Date:** 7/12/2016

Reviewer name: Lauren Dobson, Ph.D.

Reviewer Comments to Author:

On page 1 in the data description line 59 the authors state the "Wisent is now listed as threated", perhaps threatened is the word to use.

Why was the tongue used for genetic material?

This is an informative paper on the wisent assembly. However, more comparison to the yak and cattle genome could be informative on the assembly that was done. For instance comparing, N50 sizes and then also the predicted number of genes for the wisent. This could verify that the assembly is complete and thorough by using this comparison.

Level of Interest

Please indicate how interesting you found the manuscript: An article whose findings are important to those with closely related research interests

Quality of Written English

Please indicate the quality of language in the manuscript: Needs some language corrections before being published

Declaration of Competing Interests

Please complete a declaration of competing interests, considering the following questions:

- Have you in the past five years received reimbursements, fees, funding, or salary from an organisation that may in any way gain or lose financially from the publication of this manuscript, either now or in the future?
- Do you hold any stocks or shares in an organisation that may in any way gain or lose financially from the publication of this manuscript, either now or in the future?
- Do you hold or are you currently applying for any patents relating to the content of the manuscript?
- Have you received reimbursements, fees, funding, or salary from an organization that holds or has applied for patents relating to the content of the manuscript?
- Do you have any other financial competing interests?
- Do you have any non-financial competing interests in relation to this paper?

If you can answer no to all of the above, write 'I declare that I have no competing interests' below. If your reply is yes to any, please give details below.

I declare that I have no competing interests

I agree to the open peer review policy of the journal. I understand that my name will be included on my report to the authors and, if the manuscript is accepted for publication, my named report including any attachments I upload will be posted on the website along with the authors' responses. I agree for my report to be made available under an Open Access Creative Commons CC-BY license (http://creativecommons.org/licenses/by/4.0/). I understand that any comments which I do not wish to be included in my named report can be included as confidential comments to the editors, which will not be published.

I agree to the open peer review policy of the journal
