# Supplementary material for: The genome sequence of the wisent (Bison bonasus)
Source: Gigascience. 2017 Mar 10;6(4):1–5. doi: 10.1093/gigascience/gix016 (PMC5530314; doi:10.1093/gigascience/gix016)
Supplement: Reviewer_3_Report_(Revision_1).pdf [file gix016_Reviewer_3_Report_(Revision_1).pdf]

Reviewer Report

Title: "The genome sequence of the wisent (Bison bonasus)"

Version: Revision 1 **Date:** 11/8/2016

Reviewer name: Lauren Dobson, Ph.D.

Reviewer Comments to Author:

Summary: Wang et al. provide a detailed report on their assembly of the wisent genome sequence. The authors go into detail on the assembly of Illumina sequenced reads and subsequent annotation for the wisent. The comparisons to other mammalian species to the information obtained from the wisent genome is useful in determining Bovidae phylogenetic relationships. However, this paper still has some issues that need to be addressed:

Figure S6: I believe the one of the labels in the figure should be "Intron" and not "Introl length"

Table S1: The column label is "sequence coverate" and should be "sequence coverage"

Page 1

Abstract ln 21: "genomes reproduces the close relationship of bison and wisent" not if bison and wisent

Abstract1 ln 30-34: This sentence needs to be restated to either "These results will be" or "The results will be" and the "evolutionary history of the bovine species" is still not correct.

Data Description Background ln 46: "Wisent is an impressively mammal" perhaps "impressive" or another term

Page 2

Sequencing ln 2: How old and when was the wisent sample obtained? The authors state in the abstract that the wisent was rescued 80 years ago from 12 founding individuals so some confusion could occur that they sampled a wisent from 80 years ago and was one of the 12 founding animals. I do not think that was the case but more background on the sample would be nice.

Sequencing ln 15: A word seems to be missing that connects the "genomic DNA was … and circularized"

Assembly ln 34: Is it supposed to be >2kb or >2kp?

Page 4:

Ln 6: Compared the gene set of" needs to be changed to "Comparison of the gene sets of"… showed there were no…

Ln 15 "we found no pathway show significant difference" should be no pathways

Ln 18-19: "contain significant more genes in yak" should change to "contain significantly more…"

Ln 21: "and the gene composition were conservative in these three species" change conservative to conserved.

Comparative Analysis ln 26: "We downloaded the nuclear genomes of including yak…." Remove the "of including" and change to for

Comparative Analysis ln 31: add space between spanning and 1,055 Mb.

Comparative Analysis ln 40: still has bovine species

Clarification on the animal chosen to represent the wisent genome is important not only to better understand when the sample was collected but what that could mean for what is found in the genome. As with North American bison, wisent can breed with domestic cattle and create hybrids that are hard to distinguish if back crossed by just looking at them. What precautions were taken to ensure that the animal chosen was not a hybrid, with either cattle, or North American bison?

Level of Interest

Please indicate how interesting you found the manuscript: An article of importance in its field

Quality of Written English

Please indicate the quality of language in the manuscript: Needs some language corrections before being published

Declaration of Competing Interests

Please complete a declaration of competing interests, considering the following questions:

- Have you in the past five years received reimbursements, fees, funding, or salary from an organisation that may in any way gain or lose financially from the publication of this manuscript, either now or in the future?
- Do you hold any stocks or shares in an organisation that may in any way gain or lose financially from the publication of this manuscript, either now or in the future?
- Do you hold or are you currently applying for any patents relating to the content of the manuscript?
- Have you received reimbursements, fees, funding, or salary from an organization that holds or has applied for patents relating to the content of the manuscript?
- Do you have any other financial competing interests?
- Do you have any non-financial competing interests in relation to this paper?

If you can answer no to all of the above, write 'I declare that I have no competing interests' below. If your reply is yes to any, please give details below.

I declare that I have no competing interests

I agree to the open peer review policy of the journal. I understand that my name will be included on my report to the authors and, if the manuscript is accepted for publication, my named report including any attachments I upload will be posted on the website along with the authors' responses. I agree for my report to be made available under an Open Access Creative Commons CC-BY license (http://creativecommons.org/licenses/by/4.0/). I understand that any comments which I do not wish to be included in my named report can be included as confidential comments to the editors, which will not be published.

I agree to the open peer review policy of the journal
