# Supplementary material for: The genome sequence of the wisent (Bison bonasus)
Source: Gigascience. 2017 Mar 10;6(4):1–5. doi: 10.1093/gigascience/gix016 (PMC5530314; doi:10.1093/gigascience/gix016)
Supplement: Reviewer_4_Report_(Original_Submission).pdf [file gix016_Reviewer_4_Report_(Original_Submission).pdf]

Reviewer Report

Title: "The genome sequence of the wisent (Bison bonasus)"

Version: Original Submission **Date:** 7/19/2016

Reviewer name: Zhihua Jiang

Reviewer Comments to Author:

Comments on Daft genome of European bison (wisent), Bison bonasus by Wang et al.

Wang and colleagues report the first, but the draft genome of Europen bison. The material was collected from a dead male animal for DNA extraction. Both long and short size libraries were prepared and sequenced using the Illumina platform. As results, the authors were able to assemble 2.58 Gb of sequences for the species with over 120x of sequencing depth. Overall, this paper presents a simple data report.

Like cow and yak, the America bison was found to have 60 chromosomes (http://www.wow.com/wiki/List_of_number_of_chromosomes_of_various_organisms). How about the European bison then? As such, can authors map these scaffolds to chromosomes?

Several draft genomes of ruminant species are available. Can this work fill some gaps in determination of evolutionary relationships among bovini?

Did the authors find any species-specific genes in wisent? Any transcriptome data to support those non-coding genes discovered in the study? How many non-coding sequences were derived from the coding genes?

Are there any reads related to the mitochondrial genome in the species?

Can the authors compile a list of potential SNPs for the species?

What does this work contribute to genome sciences and communities?

Level of Interest

Please indicate how interesting you found the manuscript: An article whose findings are important to those with closely related research interests

Quality of Written English

Please indicate the quality of language in the manuscript: Needs some language corrections before being published

Declaration of Competing Interests

Please complete a declaration of competing interests, considering the following questions:

- Have you in the past five years received reimbursements, fees, funding, or salary from an organisation that may in any way gain or lose financially from the publication of this manuscript, either now or in the future?
- Do you hold any stocks or shares in an organisation that may in any way gain or lose financially from the publication of this manuscript, either now or in the future?
- Do you hold or are you currently applying for any patents relating to the content of the manuscript?
- Have you received reimbursements, fees, funding, or salary from an organization that holds or has applied for patents relating to the content of the manuscript?
- Do you have any other financial competing interests?
- Do you have any non-financial competing interests in relation to this paper?

If you can answer no to all of the above, write 'I declare that I have no competing interests' below. If your reply is yes to any, please give details below.

I declare that I have no competing interests.

I agree to the open peer review policy of the journal. I understand that my name will be included on my report to the authors and, if the manuscript is accepted for publication, my named report including any attachments I upload will be posted on the website along with the authors' responses. I agree for my report to be made available under an Open Access Creative Commons CC-BY license (http://creativecommons.org/licenses/by/4.0/). I understand that any comments which I do not wish to be included in my named report can be included as confidential comments to the editors, which will not be published.

I agree to the open peer review policy of the journal
